# A Novel Low-Latency and Energy-Efficient Task Scheduling Framework for Internet of Medical Things in an Edge Fog Cloud System

**DOI:** 10.3390/s22145327

**Published:** 2022-07-16

**Authors:** Kholoud Alatoun, Khaled Matrouk, Mazin Abed Mohammed, Jan Nedoma, Radek Martinek, Petr Zmij

**Affiliations:** 1 Department of Computer Engineering, Faculty of Engineering, Al-Hussein Bin Talal University, Ma’an 71111, Jordan; engkholoud88@yahoo.com (K.A.); khaled.matrouk@ahu.edu.jo (K.M.); 2 College of Computer Science and Information Technology, University of Anbar, Ramadi 31001, Iraq; mazinalshujeary@uoanbar.edu.iq; 3 Department of Telecommunications, Faculty of Electrical Engineering and Computer Science, VSB-Technical University of Ostrava, 70800 Ostrava, Czech Republic; 4 Department of Cybernetics and Biomedical Engineering, Faculty of Electrical Engineering and Computer Science, VSB-Technical University of Ostrava, 70800 Ostrava, Czech Republic; radek.martinek@vsb.cz; 5 Industrial Engineering—Brose Group, Prumyslovy Park 302, 74221 Koprivnice, Czech Republic; petr.zmij@brose.com

**Keywords:** low-latency, Cardiovascular Disease, ECG sensors, fog computing, health monitoring system, internet of medical things, scheduling algorithms, task scheduling

## Abstract

In healthcare, there are rapid emergency response systems that necessitate real-time actions where speed and efficiency are critical; this may suffer as a result of cloud latency because of the delay caused by the cloud. Therefore, fog computing is utilized in real-time healthcare applications. There are still limitations in response time, latency, and energy consumption. Thus, a proper fog computing architecture and good task scheduling algorithms should be developed to minimize these limitations. In this study, an Energy-Efficient Internet of Medical Things to Fog Interoperability of Task Scheduling (EEIoMT) framework is proposed. This framework schedules tasks in an efficient way by ensuring that critical tasks are executed in the shortest possible time within their deadline while balancing energy consumption when processing other tasks. In our architecture, Electrocardiogram (ECG) sensors are used to monitor heart health at home in a smart city. ECG sensors send the sensed data continuously to the ESP32 microcontroller through Bluetooth (BLE) for analysis. ESP32 is also linked to the fog scheduler via Wi-Fi to send the results data of the analysis (tasks). The appropriate fog node is carefully selected to execute the task by giving each node a special weight, which is formulated on the basis of the expected amount of energy consumed and latency in executing this task and choosing the node with the lowest weight. Simulations were performed in iFogSim2. The simulation outcomes show that the suggested framework has a superior performance in reducing the usage of energy, latency, and network utilization when weighed against CHTM, LBS, and FNPA models.

## 1. Introduction

As technology has advanced, applications of the Internet of Things (IoT) have become a part of our everyday lives. As a result, the number of devices employed in these applications will grow, resulting in massive amounts of data being generated. Because the information will be transmitted to the cloud for processing, there will be a delay in response because the cloud is located far away from these devices. Every physical device, such as cameras, automobiles, sensors, wearables, and home appliances, is connected via IoT apps [1]. One of the core branches of IoT is ubiquitous home healthcare, also known as the Internet of Medical Things (IoMT). Recent developments in IoT and the increasing use of wearables to collect physiological data and vital signals have led to new distributed computing paradigms that combine wearables and IoMT for remote telecare [2]. Low latency, mobility, location awareness, and a fast reaction time are all requirements for many applications. For different applications of healthcare systems, a straightforward sensor-to-cloud architecture is not feasible, owing to the fact that most clinics and hospitals do not want to retain patient data externally [3]. Additionally, there is always the possibility of a crucial network or data center failure, putting patients’ lives in jeopardy [4]. Using the cloud only causes delays while transferring information from IoT devices to the cloud and from the cloud to doctors or hospitals. In the healthcare system, there must be a rapid response to emergency situations, and this requires actions in real time, where time and efficiency play an important role, and this may be harmed as a result of the cloud’s latency [5]. Thus, classic centralized cloud computing should be expanded to a distributed architecture. The term “distributed architecture” denotes the process of separating duties and subsequently offloading them to several nodes. Despite the fact that many academics have created techniques to increase the performance of cloud computing in real-time applications, IoT applications and mobile services still face several hurdles, including low latency, high reaction speed, cost, energy consumption, mobility support, and geo-distribution [6]. Thus, it was essential to create new technology that was closer to IoT devices and that could solve the cloud’s difficulties. In 2012, Cisco proposed fog computing [7].

Fog computing is a novel computing paradigm that spreads cloud computing from the network’s core to its edge. Its purpose is to increase user access to computer, storage, and networking resources [8]. Previous challenges can be dealt with by fog computing [9]. According to the prediction by Cisco, approximately 1 trillion IoT devices will be linked to the internet by 2025 [10]. Resource management in the fog computing environment has become a big concern due to changes in the devices of users in terms of bandwidth, computation, latency, and storage [11]. Tasks in a fog environment are classified into two categories: those that require computing intensity and those that require information intensity. When scheduling tasks that require a lot of computational power, the scheduler moves the data to a high-productive resource, which reduces task achievement time. Furthermore, it is sought to limit the amount of data transfer when scheduling jobs that need data intensity. As a result, the data migration time is shortened [12]. The amount of IoT equipment is currently growing, which raises the processing demand on fog and cloud nodes. As a result, an efficient technique for scheduling jobs and managing resources in fog and cloud settings is required [11]. One of the important technologies that aid the seamless operation of IoT healthcare systems and monitoring applications is fog computing. Since healthcare applications are latency-sensitive and require real-time monitoring, decision making and data analysis are critical requirements. Home nursing for the elderly and patients with chronic diseases such as heart disease, high blood pressure, diabetes, and so on is an example of this type of application [4,13].

The Internet of Medical Things (IoMT) refers to the ability to connect medical devices to monitor vital medical signals and diagnose patients’ diseases [14]. Additionally, it interconnects medical devices with healthcare providers such as hospitals, private companies, or medical researchers [15]. IoMT combines IoT with conventional medical equipment and expands sensing and capabilities [16]. IoMT has the ability to transmit data across a network without demanding human-to-human or human-to-computer interaction [14]. The emergence of IoMT is mainly due to the increased use and development of connected and distributed medical devices [15], as well as the emergence of many health applications. IoMT plays an essential and vital role in remote healthcare monitoring. One of the most important types of health monitoring is related to the heart because the measured vital signs can detect numerous diseases that are hidden in nature, such as arrhythmia [14]. Due to the critical nature of healthcare-related systems, IoMT still faces various difficulties, especially with regard to reliability, safety, and security [15]. IoMT is one feature that 5G supports. 5G decreases latency, which increases connectivity between devices (such as healthcare devices). Moreover, it improves the QoS of data in real time, which is required in IoMT. In other words, it can be said that 5G facilitates IoMT [17]. IoMT in the healthcare system has the ability to supply hospital services at home at low costs without decreasing quality [17]. IoMT architecture consists of three main stages: The first stage is to collect data and signals from the patient through biomedical health monitoring sensors. The second stage is data analysis in the fog layer. In the third stage, if the data need further analysis and processing, they are sent to the cloud. Additionally, patients’ information and their medical history are stored in the cloud [18].

Continuous health monitoring is required in the case of numerous chronic diseases, and normal examination of physiological information is essential in these cases. However, hospitalization is not required for the purposes of continuous monitoring. In addition, the hospital stay is costly and consumes patients’ precious time. Further, with the spread of the Coronavirus, the elderly must be protected and not put their lives at risk by being in the hospital for long periods of time. Individuals can be infected with the COVID-19 virus by other individuals who have the virus. It is spread by direct contact, by respiratory droplets while sneezing or coughing, or by contacting a virus-infected surface and then touching the nose, eyes, or mouth [19]. The COVID-19 virus poses a critical hazard, particularly for elderly people and individuals who have chronic diseases, such as heart disease, hypertension, and diabetes [20]. It is starting to spread among medical teams and nurses in hospitals, and this may pose a threat to the health of hospitalized patients. Therefore, continuous health monitoring at home is the best option.

According to a World Health Organization report (WHO), approx. 17.9 million people died globally from Cardiovascular Disease (CVD). A third of deaths of people under the age of 70 and 4 in 5 deaths are due to strokes and heart attacks. Regular monitoring of patients may improve patients’ health and reduce mortality. ECG monitoring systems are advanced equipment used in the healthcare sector and have evolved significantly over time [21]. According to the latest research on thirty million healthcare-related IoT apps, the information flow reaches 25,000 records per second [22]. Additionally, in intelligent cities with more information references, information flows in real time can quickly increase every second. Moreover, due to the rapid rise of IoT, the processing capability of most gadgets cannot be attained [23]. There are various problems in fog computing. One of the main significant problems is the task scheduling operation and the identification of the appropriate resource to carry out the task. The task should not be transferred a long distance for processing, which in turn increases energy consumption and delay, so choosing the appropriate resource is difficult. To address several flaws in present task scheduling algorithms, an Energy-Efficient Internet of Medical Things to Fog Interoperability of Task Scheduling Framework is proposed. We place processing closer to IoT devices to decrease latency and energy consumption by minimizing the physical distance between devices and computing nodes. Additionally, we limit the energy in IoT devices that can be consumed when transferring tasks to a far computing node. In our architecture, ECG sensors are used to monitor heart health at home in a smart city. ECG sensors send the sensed data continuously to the ESP32 microcontroller through Bluetooth Low Energy (BLE). The ECG signals are analyzed in an ESP32 microcontroller that is also connected to the fog scheduler via Wi-Fi to send the results data of the analysis (tasks). The appropriate fog node is carefully selected to execute the task by giving each node a special weight based on the expected amount of energy consumed and latency in executing this task and choosing the node with the lowest weight.

The main contributions of this study can be summarized as follows:

1. It suggests an Energy-Efficient Internet of Medical Things to Fog Interoperability of Task Scheduling (EEIoMT) Framework for real-time healthcare applications.
2. It proposes an efficient task scheduling algorithm that increases proficiency and quality of service (QoS) and reduces energy consumption in the system and the latency for healthcare tasks.
3. The proposed solution deploys IoT services on accessible edge devices to enhance the continuous monitoring of chronic patients, improve their quality of life, and lower medical system expenses by exploiting the processing and storage capabilities at the network’s edge.

The arrangement of this study is organized into the following sections: Section 2 explores the related work on scheduling in fog computing and health monitoring systems. Section 3 presents the research methodology of the EEIoMT framework with the suggested algorithm. Section 4 shows the experimental results’ evaluation and a comparison with state-of-the-art methods. Section 5 concludes the study and introduces future work.

## 2. Related Work

Most of the related studies that focus on task scheduling in fog computing and healthcare monitoring are examined in this part to highlight the contributions and compare the outcomes with the suggested framework.

Paul et al. [4] introduced a three-layer architecture to monitor patient health using cloud and fog computing. Cloud computing servers, fog computing servers and resources, and sensors such as biomedical health monitoring sensors make up this architecture. The fog tier performs the aggregation and analysis of data that are obtained from edge devices. It then uses a task scheduling algorithm to deliver tasks to the cloud and fog nodes. The proposed algorithm and architecture were assessed as regards network use, delay, and power usage by comparing simulation results to the cloud design alone. Hassan et al. [24] presented a three-layer distant pain tracking system design, in which the fog node uses digital signal processing techniques to detect pain. The proposed solution had lower latency than a cloud-only implementation. The proposed method, however, will not be sufficient when the number of patients rises because the hospital’s data are handled by a single fog node. Abdelmoneem et al. [25] suggested a cloud-based, interoperable healthcare IoT architecture to reduce application latency and costs and meet time constraints. To achieve an effective balance in the distribution of healthcare tasks, a task scheduling and allocation approach was proposed. A variety of tasks and cloud nodes were used to evaluate the proposed system’s performance. Fog nodes are responsible for executing computational tasks such as context management and data analysis. The scheduling module represents a mapping among tasks represented as a binary graph that will be executed on fog nodes and cloud servers. The task dispatcher module assigns the scheduled tasks to fog nodes or the cloud. In terms of mass ratio, delay, and cost, the simulation’s results were satisfactory. However, it lacks necessary QoS metrics in healthcare applications, including energy consumption, processing time, and memory usage. Mukherjee et al. [26] presented a three-layer mobility-aware Internet of Health Things (IoHT) architecture that included sensor nodes, fog nodes for parametric health control, and a cloud server for evaluating aberrant health conditions. The performance of the suggested algorithm and architecture in terms of latency and power use was evaluated by comparing simulated results to the cloud-only architecture.

Mutlag et al. [27] introduced the Multi-Agent Fog Computing (MAFC) concept for managing important healthcare tasks. This strategy allocates fog resources by allowing for task prioritization at two levels: locally on fog nodes and globally in the cloud. To optimize scheduling for critical tasks, this model maps between three tables: task priorities, network load, and network resources. Then, it assigns tasks according to these tables. However, numerous criteria are required to process critical and emergency tasks. In terms of energy consumption, managed services, and latency, the proposed model’s performance was assessed by comparing simulation results to the cloud-only architecture. Ying Wah et al. [28] proposed a system relying on the fog–cloud paradigm for healthcare applications. This system implemented a novel algorithm, called Health Care-awareness Cost-Efficient Task Scheduling (HCCETS), which defines vital tasks of a heartbeat application to schedule and execute them with the lowest cost while meeting their deadline. To acquire the cost-efficiency of tasks during allocation under the requirements of QoS, the method is implemented in several phases: task prioritization, resource search, and task scheduling. The outcomes indicated that the performance of the proposed algorithm exceeded that of previous algorithms in terms of cost. However, it does not consider resource limitations or energy consumption. Additionally, this system does not support mobility awareness. Asghar et al. [29] presented a fog-relaying architecture for a health tracking system to reduce network usage and latency. When the health monitoring system is widely deployed, they also suggested a novel Load Balancing Scheme (LBS) to help fog nodes manage their load. They performed large-scale simulations in the iFogSim toolkit to validate the efficacy of the proposed technique, comparing the results to the cloud-only architecture, the LoAd Balancing (LAB) scheme, and Fog Node Placement Algorithm (FNPA) in terms of network use and latency. In comparison to cloud-only, LAB, and FNPA, the suggested health monitoring solution dramatically reduced network usage and latency. However, it does not consider energy consumption. The Fog Node Placement Algorithm (FNPA) was presented by Tun and Paing [30], which connects IoT devices to the closest fog node with sufficient resources (bandwidth, RAM, and CPU). FNPA outperformed the cloud-only implementation and the fog node with a minimal distance method by significantly reducing latency, implementation cost, and network usage. The Critical Healthcare Task Management (CHTM) model was developed by Mutlag et al. [31] and tested using an ECG dataset. At the fog level, they also established a resource scheduling model for fog nodes. They also demonstrated a multi-agent system that can manage the entire network from the edge to the cloud. To successfully manage crucial activities, the proposed solution exceeds the restrictions of interoperability, scheduling, resource sharing, and dynamic job assignment. When compared to the cloud-only model, the simulation outcomes indicated that their model decreased the time response by 90%, network usage by 79%, cost by 80%, network latency by 65%, and energy usage by 81%.

The importance of the location where the task is to be examined in the fog computing architecture was not taken into account by the task scheduling algorithms in most related publications. The incoming tasks from the IoT device were assigned to the nearest accessible fog node in these cases, regardless of the node’s capacity. Further, several of them failed to examine the task’s requirements and importance. In the evaluation of the proposed architectures, several publications never addressed the performance metric of energy consumption. Furthermore, the performance of some proposed architectures and algorithms was assessed by contrasting simulation outcomes with cloud-only architectures but not with fog-based architectures. In contrast to previous research, we ran extensive simulations using four distinct topology configurations and compared the outcomes to the best three fog-based implementations: CHTM, LBS, and FNPA models. The proposed EEIoMT outperformed the CHTM [31], LBS [29], and FNPA [30] models in four parameters: latency, energy consumption, network usage, and CPU usage.

Many researchers have developed fog-based designs for health monitoring systems that outperform cloud-based architectures. However, for the previously presented health observing systems, none of the scholars contrasted their suggested technique with a fog-based architecture. As a result, developing a fog-based strategy that is more effective than previous fog-based solutions is desirable. Because latency, energy consumption, and network usage are all significant characteristics for health monitoring systems, most studies simply compared their proposed approach to latency and network utilization. Energy consumption, however, was not regarded as a performance indicator. As a result, in addition to delay and network utilization, we must compare our suggested strategy in terms of energy consumption.

In the proposed algorithm, the tasks are classified according to the patient’s condition into normal, moderate, and critical. Then, critical tasks are given high priority, moderate tasks are given medium priority, and normal tasks are given low priority so that each classified task is placed in its own queue using the Earliest Deadline First (EDF) algorithm. After that, the appropriate fog node is carefully selected to execute the task by giving each fog node a special weight based on the expected amount of energy consumed and latency in executing this task and choosing the lowest weight.

## 3. Research Methodology

**Figure 1:**
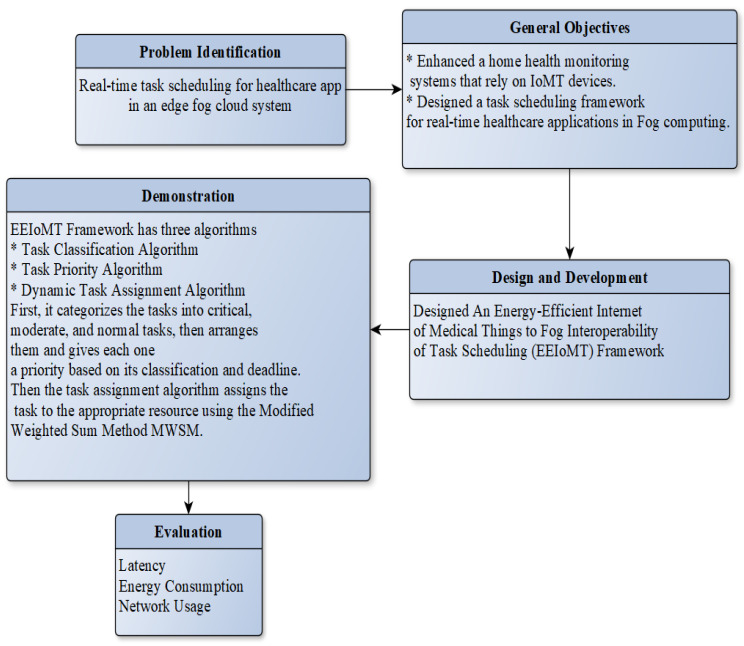
Research Methodology.

This part presents the methodology of the EEIoMT framework. Figure 1 depicts the research methodology flowchart. In order to develop a solution, we first looked at previous studies to determine the most pressing issue and the elements that influence it. The second step was to determine the goals. The proposed framework’s design and development was the third step. The role of each algorithm in the framework to tackle the problem was identified during the demonstration process. The final step was to analyze the outcomes by evaluating energy consumption, latency, and network usage with the iFogSim2 simulator.

### 3.1. Proposed Framework Architecture

The proposed architecture assumes that a group of patients is being monitored in a smart city for emergency situations such as arrhythmia. Cardiovascular disorders are well-known to be among the top main causes of death in the world today. Cardiologists utilize the ECG sensor as one of the tools to diagnose and identify indicators of heart disease in their patients because preserving patients’ lives from a sudden heart attack or cardiac arrest necessitates quick and correct interpretation and decisions. As a result, patients’ current conditions, emergency situations, and decisions are addressed to the best of their abilities. Two important and fundamental design factors related to the scope of this research must be considered: First, the healthcare system must operate 24/7 without downtime. Thus, the caregiver can always monitor the condition of the patients. Second, the information must always be up to date. If the information is not up to date, the missing factors could cost human lives. Furthermore, in comparison to static techniques in the literature, our system allows for real-time dynamic allocation of health duties across fog and cloud devices based on the algorithms employed.

**Figure 2:**
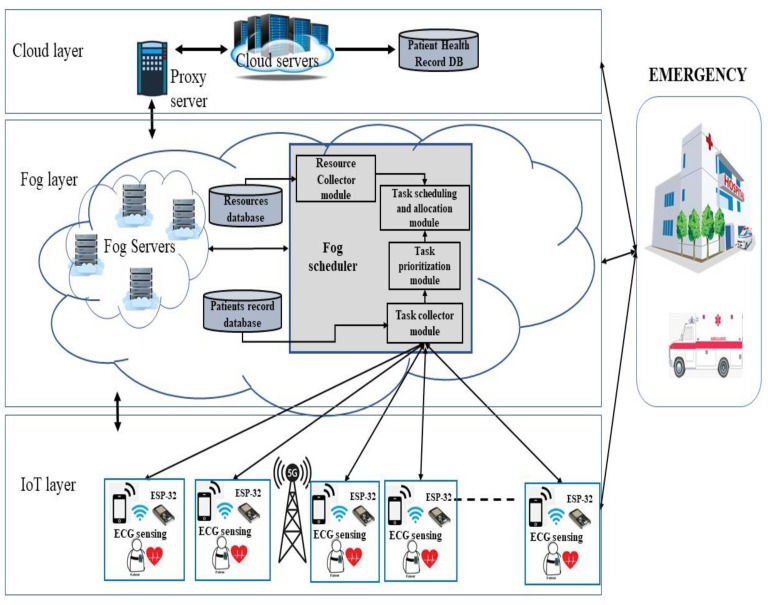
The proposed EEIoMT framework.

The architecture is made up of three layers: a bottom layer (IoT and edge equipment), an intermediate computing layer (fog devices), and a top layer (cloud). The IoT device is the lowest layer, and it comprises all of the connected devices that are involved in the sensing and actuation processes. For time-sensitive task applications, task achievement should be performed by the appropriate compute node. Because no standard architecture is available at this time, one of the current designs must be chosen and modified to make it appropriate for the algorithm to be created. For this study, a three-tier architecture is used, as presented in Figure 2. The update aims to reduce latency in real-time data transfer while simultaneously improving QoS.

The following are the detailed three-tier layer architectures.

- First tier (IoT devices and edge devices): The first layer is a collection of sensors, actuators, and ESP32 microcontrollers. Actuators can be a smartphone or smartwatch. In the proposed architecture, ECG sensors are used to evaluate heart health and send the sensed data continuously to the ESP32 microcontroller through Bluetooth (BLE). This microcontroller is responsible for: (1) data acquisition: it collects the ECG signals from the ECG sensor; (2) data analysis: it analyzes the ECG signals and classifies the patient condition into normal or abnormal in comparison with standard ECG intervals. The microcontroller is connected to the fog scheduler through Wi-Fi. The smartphone is used to receive a notification from the system or from the specialist doctor.
- Second tier (fog layer): This layer is set near the end gadgets. It consists of fog servers and the fog scheduler. Fog devices include computation, communication, and storage capabilities, as well as the ability to perform a variety of processing tasks. The fog scheduler is linked to a network of IoT devices that typically spans a neighborhood or a small town, and it performs task analysis in real time. Each fog device has an internal database that is used to compute and store the tasks that have been assigned to it. In addition to the internal databases, this layer maintains two global fog databases: (1) a resource database, which reports on the capability for processing, usage, remaining energy, and each processor device’s storage, and (2) a patient record database, which stores patient health data and analysis results. The fog scheduler is in charge of accessing information from these databases, aiming to manage the scheduling of those resources.

The fog scheduler includes the following modules: (1) The Task Collector Module collects all tasks from the ESP32 microcontrollers that need to be processed. It also classifies the tasks according to the proposed task classification algorithm. It creates a series of structures that contain information and task characteristics. (2) The Task Prioritization Module prioritizes the tasks based on the task priority algorithm. Critical tasks are given high priority, moderate tasks are medium priority, and normal tasks are low priority. There is a queue for each task classification; the tasks are arranged in each queue based on their deadlines. As a result, critical activities will be completed sooner, thus helping to satisfy the deadline and reaction time requirements. (3) The Resource Collector Module receives information about the fog layer’s available resources. It keeps track of how often the fog devices are used, as well as their updated capabilities (processor, RAM, energy, etc.). (4) The Task Scheduling and Allocation Module is in charge of establishing and customizing the task allocation method for fog and cloud devices. This module principally provides a software routine for an optimization method aimed at lowering the system’s energy consumption and decreasing the time that it takes to complete health tasks.

- The cloud, which gathers data and information from intermediary computing nodes, is the third tier (cloud layer) of the architecture (fog layer). It uses cloud-based resource devices to complete portions of jobs that are not completed in the fog layer, which are typically non-time-sensitive. It also has a permanent database that caregivers can use to keep track of the patient’s health analysis results. A proxy server establishes a communication channel between a cloud server and fog nodes.

The suggested architecture has been utilized to enable rapid response, IoT healthcare, and monitoring applications since these applications are latency-sensitive and require real-time monitoring. In healthcare applications, information analysis and decision making are critical. The most time-sensitive data (critical tasks) are processed in the appropriate fog node in our architecture; this allows important jobs to be completed earlier, which helps in meeting the deadline and response time requirements.

### 3.2. The Proposed Task Scheduling Algorithm

Various sensors and equipment provide huge amounts of data in real-time applications, such as healthcare applications, which involve crucial tasks. Fog computing implementation can be used to manage them at the network’s edge. However, fog nodes lack resources, which may limit the amount of time required for the ultimate outcome/analytics. Only a few tasks can be performed by fog nodes. Choosing which tasks the fog nodes will perform is a difficult decision.

To address various flaws in existing task scheduling algorithms, an Energy-Efficient Internet of Medical Things to Fog Interoperability of Task Scheduling (EEIoMT) framework is proposed. This framework has three algorithms: a task classification algorithm to classify the tasks into three categories (normal tasks, moderate tasks, and critical tasks), a task priority algorithm to give priority to each task according to each category from the previous algorithm, and a dynamic task assignment algorithm to allocate tasks to the proper fog node. The major function of algorithms in this framework is to assign tasks and prioritize them, as well as to determine network resource availability. We place processing nodes closer to sensors to reduce latency and energy consumption by minimizing the physical distance between sensors and computing nodes. We also limit the energy in IoT devices that can be consumed when transferring tasks to a far computing node. Each task should be completed as quickly as possible to save the patient’s life, such as contacting the civil defense and the hospital.

#### 3.2.1. Mathematical Model for Task Scheduling Algorithm

There are three layers of processing in the suggested framework: ESP32 microcontrollers, fog nodes, and the cloud. Fog servers, which are micro data centers and virtual machines, are present in each node in the fog layers. Each layer’s server capacity is different. Fog nodes have substantially less computational power, storage, and server capacity than the fog cloud, and fog nodes also have significantly more computing power, storage, and capacity than the ESP32 microcontroller. However, when it comes to delays, response time, and distance from end users, the closer the computing nodes are to the data source, the lower the delay and the higher the response speed. Therefore, critical and time-sensitive tasks must be executed at the appropriate computational node to ensure that they are executed with minimal delay and meet their deadlines.

In fog computing, the task scheduling challenge is to allocate IoT tasks to suitable fog nodes from a list of potential fog nodes in order to optimize QoS. In this research, latency, energy consumption, and network usage are considered QoS parameters.

Assuming that there are *n* tasks T to be delivered to the fog scheduler, the following can be expressed:

(1) $$T=\left\{t_{1},\;t_{2},\;\ldots ,\;t_{n}\right\}$$

in which each parameter $t_{i}$ is characterized using a set of attributes $t_{i}=\left\{TS_{i},\;TL_{i},\;type_{i},\;dt_{i}\right\}$, where $TS_{i}$ is task size (in bits), $TL_{i}$ is task length (in MI (Millions of Instructions)), the $type_{i}$ of the task is normal, moderate, or critical, and $dt_{i}$ is the deadline of the task to be respected for achieving the task.

Assume that the fog computing system has m Fog nodes F, which may be expressed as follows:

(2) $$F=\left\{f_{1},f_{2},\;\ldots ,\;f_{m}\right\}$$

in which each parameter $f_{j}$ is described using a set of attributes $f_{j}=\left\{S_{j},\;CC_{j},\;E_{j}\right\}$, where $S_{j}$ refers to storage capacity, $CC_{j}$ is computing capacity (in MIPS (Millions of Instructions Per Second)), and $E_{j}$ is the total battery capacity (Energy) of $f_{j}$.

The task scheduling challenge is to allocate n tasks to m fog nodes in such a way that the QoS parameters are optimal using the notations above. $X_{ij}$ refers to the assignment of task $t_{i}$ to fog node $f_{j}$, whereas $X_{icloud}$ refers to the assignment of task $t_{i}$ to the cloud.

We can analytically analyze the execution time, transmission time, response time, and energy use to distribute the requested tasks to the appropriate fog node. After that, the relevant node is chosen, and the task is assigned to it.

- Execution time

The execution time of processing task $t_{i}$ on fog node $f_{j}$ or the cloud is calculated according to Equations (3) and (4):

(3) $$Et\left(X_{ij}\right)=\frac{TL_{i}}{CC_{j}}$$

(4) $$Et\left(X_{icloud}\right)=\frac{TL_{i}}{CC_{cloud}}$$

where $Et\left(X_{ij}\right)$, and $Et\left(X_{icloud}\right)$ are the execution time of the task in the fog node and cloud, respectively, and $CC_{j}$, and $CC_{cloud}$ are the computation capacity of the fog node, and cloud, respectively.

- Transmission time

The transmission time of task $t_{i}$ from the ESP32 to the fog scheduler $T_{r}t\left(FS\right)$ is calculated by the task size $TS_{i}$ of task $t_{i}$ divided by the transmission rate (bandwidth) BW.

(5) $$T_{r}t\left(FS\right)=\frac{TS_{i}}{BW}$$

The transmission time of task $t_{i}$ from the fog scheduler to the fog node $T_{r}t\left(X_{ij}\right)$ or to the cloud $T_{r}t\left(X_{icloud}\right)$ is calculated as follows:

(6) $$T_{r}t\left(X_{ij}\right)=\frac{TS_{i}\;send+TS_{i}\;response}{BW}$$

(7) $$T_{r}t\left(X_{icloud}\right)=\frac{TS_{i}\;send+TS_{i}\;response}{BW}$$

The total transmission time of tasks from the ESP32 to the appropriate fog node $f_{j}$ or the cloud can be calculated by combining Equations (5) and (6) or (5) and (7), respectively.

(8) $$T_{r}t_{total}\left(X_{ij}\right)=T_{r}t\left(FS\right)+T_{r}t\left(X_{ij}\right)$$

(9) $$T_{r}t_{total}\left(X_{icloud}\right)=T_{r}t\left(FS\right)+T_{r}t\left(X_{icloud}\right)$$

- Response time

The response time of a task $t_{i}$ that is handled in many layers is the sum of the specified node’s execution time plus the task’s transmission time from the source to the destination. The next formula is utilized to compute the response time of task $t_{i}$ that is handled at fog node $f_{j}$:

(10) $$RT\left(X_{ij}\right)=Et\left(X_{ij}\right)+T_{r}t_{total}\left(X_{ij}\right)$$

The response time of task $t_{i}$ that is processed in the cloud is calculated as follows:

(11) $$RT\left(X_{icloud}\right)=Et\left(X_{icloud}\right)+T_{r}t_{total}\left(X_{icloud}\right)$$

- Energy consumption

Energy consumption in our work is composed of two components: the energy spent on transmitting a task to a computing node and the energy spent on executing the task.

The energy $E_{tr}\left(X_{ij}\right)$ required to transmit task $t_{i}$ to fog node $f_{j}$ is calculated by multiplying the transmission time by a constant coefficient as follows:

(12) $$E_{tr}\left(X_{ij}\right)=\lambda *T_{r}t_{total}\left(X_{ij}\right)$$

However, when calculated, the energy required to transmit task $t_{i}$ to the cloud is calculated as follows:

(13) $$E_{tr}\left(X_{icloud}\right)=\lambda *T_{r}t_{total}\left(X_{icloud}\right)$$

where λ is a constant related to the wireless interface [32]. The energy consumption $E_{p}\left(X_{ij}\right)$ for processing task $t_{i}$ on fog node $f_{j}$ is expressed by multiplying the execution time by a constant coefficient as follows:

(14) $$E_{p}\left(X_{ij}\right)=\mu *Et\left(X_{ij}\right)$$

The energy consumption $E_{p}\left(X_{icloud}\right)$ for processing task $t_{i}$ in the cloud is as follows:

(15) $$E_{p}\left(X_{icloud}\right)=\mu *Et\left(X_{icloud}\right)$$

where μ is the coefficient denoting the energy consumption per CPU cycle [33].

The total energy is calculated by a combination of processing energy and transmission energy for the fog or cloud, as follows:

(16) $$E_{total}\left(X_{ij}\right)=E_{tr}\left(X_{ij}\right)+E_{p}\left(X_{ij}\right)$$

(17) $$E_{total}\left(X_{icloud}\right)=E_{tr}\left(X_{icloud}\right)+E_{p}\left(X_{icloud}\right)$$

- Makespan

The makespan is the maximum completion time (CT) of a resource. It is denoted as follows, according to the equation in [34]:

(18) $$Makespan=MAX\;\left(\;\overset{n}{\underset{i=1}{\sum }}CT_{i,j}\;\right)$$

- Resource Utilization

Resource Utilization (RU) refers to the most efficient use of resources. Reduced makespan is critical to utilization performance. As a result, these two concepts are inversely connected. The usage of Virtual Machines (VMs) is computed as follows:

(19) $$RU=\frac{CT_{i,j}}{Makespan}$$

The total use of all assets present in the fog environment is defined as the average utilization of the resource. The average resource consumption is computed as follows:

(20) $$ARU=\overset{n}{\underset{i=1}{\sum }}RU$$

- Proposed formulation for task scheduling problem

The goal of scheduling is to distribute IoT tasks to the resources of fog nodes or the cloud in the most efficient way possible to reduce latency and energy consumption. Integer Linear Programming (ILP) [35] can be used to represent the issue of assigning IoT tasks to suitable fog nodes, as follows:

(21) $$\overset{m}{\underset{j=1}{\sum }}X_{i,j}=1\;\;\;\;\;\;\;\;\;\;\forall i\;\in \left\{1,\ldots ,n\right\};$$

(22) $$\overset{n}{\underset{i=1}{\sum }}X_{i,j}\times C_{i}\leq \;CC_{j}\;\;\;\;\;\;\;\;\;\forall j\;\in \left\{1,\ldots ,m\right\};$$

(23) $$\overset{n}{\underset{i=1}{\sum }}X_{i,j}\times TS_{i}\leq \;S_{j}\;\;\;\;\;\;\;\;\forall j\;\in \left\{1,\ldots ,m\right\};$$

(24) $$\overset{n}{\underset{i=1}{\sum }}X_{i,j}\times E_{p}\left(X_{ij}\right)\leq \;E_{j}\;\;\;\;\;\forall j\;\in \left\{1,\ldots ,m\right\};$$

(25) $$RT\left(X_{ij}\right)\leq dt_{i}\;\;\;\;\;\;\;\;\;\forall i\;\in \left\{1,\ldots ,n\right\},\;\forall j\;\in \left\{1,\ldots ,m\right\}$$

(26) $$X_{ij}\in \left\{0,1\right\}\;\;\;\;\;\;\;\;\;\;\;\;\;\;\;\forall i\;\in \left\{1,\ldots ,n\right\},\;\forall j\;\in \left\{1,\ldots ,m\right\}$$

Equation (21) prevents a task from being assigned to more than one fog node at the same time. Equation (22) shows that the computation intensity necessary to complete a set of tasks assigned to the fog node cannot be greater than the computing capability of the fog node. Equation (23) states that the task size necessary to complete a set of tasks given to the fog node cannot exceed the fog node’s storage capacity. Equation (24) ensures that the fog node’s energy consumption needed for completing a set of activities is less than the fog node’s remaining battery capacity. Equation (25) ensures that the total time required by fog node $f_{j}$ to complete task $t_{i}$ does not exceed the task deadline. Equation (26) defines our binary decision variables, in which $X_{ij}$ is 1 if $f_{j}$ is selected for performing task $t_{i}$ and 0 otherwise.

The fog scheduler selects the correct fog node by assigning a weight to each node so that the selection ensures that the optimization of QoS parameters is achieved. We modified the Weighted Sum Method (WSM) to be the dynamic Modified WSM (MWSM) and compatible with our work.

(27) $$N_{ij}^{MWSM-score}=w_{E}*\;E_{total}\left(X_{ij}\right)+w_{L}*\;RT\left(X_{ij}\right)\;\;\;\;\;for\;j=\left\{1,2,\ldots ,\;m\right\}$$

where $N_{ij}^{MWSM-score}$ indicates the QoS score of $X_{ij}$ (the weight assigned to fog node $f_{j}\;)$. The lower the $N_{ij}^{MWSM-score}$, the more appropriate the fog node for executing task $t_{i}$. The QoS score given to $X_{ij}$ is calculated as the weighted sum of QoS parameters, total energy consumption, and latency (expressed as response time). The summation of the weight factors must be equal to 1, as shown below.

(28) $$w_{E}+w_{L}=1\;\;$$

where $w_{E}$ and $w_{L}$ are weight factors relating to the significant influence of the QoS parameters energy consumption and latency in a given node, respectively.

Nomenclature contains a list of all of the notations used in the mathematical model.

#### 3.2.2. ECG Signal Analysis

**Figure 3:**
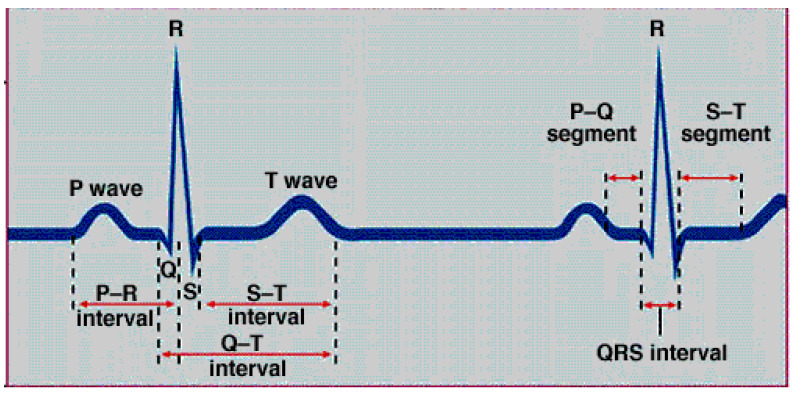
ECG wave and intervals [37].

The diagnosis of cardiac problems known as arrhythmias, such as bradycardia, tachycardia, and heart rate variation, is made easier with an ECG feature extraction algorithm. The heart rate and arrhythmias are determined using beat detection, and abnormal beats are detected using additional processing. The acquired ECG signal can be segmented into the P wave, T wave, and QRS complex. Different time intervals, such as PR, QRS, QT, and ST, are obtained, as shown in Figure 3.

The ECG signals are analyzed every 8 s [36] and classify the patient’s state into normal or abnormal in comparison with the typical values of waves and intervals of ECG signals, which are shown in Table 1. If the result of analyzing the ECG signals is within the permissible duration for each wave or interval, the patient’s condition is considered normal. However, if there is any difference, whether an increase or decrease in the periods, the patient has an abnormal condition and must be monitored.

In our work, the Novel Windowing Algorithm [38] was used to analyze ECG signals in the ESP32 microcontroller.

#### 3.2.3. Task Classification Algorithm

We classified IoT tasks into three types: normal tasks, moderate tasks, and critical tasks according to the patient’s condition.

- Normal tasks

If the condition of the patient is normal, the tasks are considered normal tasks. The data will be aggregated in ESP32, and the analysis will be performed every 24 s, for example, instead of 8 s, aiming to decrease energy usage during the analysis. In the given period of time, the patient’s health data will be delivered to the patient record database in the fog layer to update and store their data.

- Moderate tasks

If the patient’s condition is abnormal and the patient has no history of heart attacks, the tasks are considered moderate tasks. These tasks are delivered to the fog scheduler to be treated in the appropriate fog node.

- Critical tasks

If the patient’s condition is abnormal and the patient has a history of heart attacks, the tasks are considered critical tasks. These tasks must be processed in the appropriate available fog node because they are time-sensitive, and delays in response may put the patient’s life at risk.

#### 3.2.4. Task Priority Algorithm

To assign tasks to the correct fog node and ensure that tasks are executed by their deadline, tasks must be scheduled according to their priority. In the priority algorithm, critical tasks are given high priority and should be treated in the appropriate fog node. Although moderate tasks have a medium priority, when the deadline for these tasks approaches, moderate tasks will be changed to critical tasks, and they will be given a high priority. Normal tasks are not time-sensitive tasks, so these tasks have low priority.

#### 3.2.5. Dynamic Task Assignment Algorithm

After categorizing the tasks and giving each of them a priority, it is necessary to select the correct fog node for processing. When selecting a fog node, we make sure that the QoS is optimized in terms of lag and energy consumption. A dynamic task assignment algorithm is proposed to assign tasks dynamically to the correct fog node according to the task priority, the weight of the fog node, and resource availability.

The Weighted Sum Method (WSM) [40] is a multi-criterion decision-making method that takes into account a list of criteria, such as current battery state, energy consumption, latency, response time, and load, among others. The WSM is the best-known and most widely used method for making decisions with multiple criteria. The method is clearly explained in [41]. We can make a decision using this technique based on a lot more useful data on the effectiveness of any node. Each node has a different weight, which allows it to stand out from the others. The WSM is time-independent and can be described as a static WSM. Therefore, we modified this method to be a dynamic MWSM and compatible with our work.

The MWSM is implemented to select the appropriate node in terms of latency and energy consumption according to Equation (27) in the mathematical model.

If the task is critical, the weight factors are $w_{E}=0;\;w_{L}=1$. This type of task is time-sensitive, so the task should be executed with the least possible latency because any further delay will put the patient’s life at risk. When the task is moderate, the $weight\;factors\;w_{E}=0.5;\;w_{L}=0.5$ are used. In order to conserve energy and, at the same time, take care of the delay, we set an equal weight factor between them while ensuring the execution of these tasks within their deadlines. However, in the case of normal tasks, the $weight\;factors\;w_{E}=1;\;w_{L}=0$ are used. These tasks are not time-sensitive, and therefore, we do not consider the latency and focus on energy while ensuring the execution of these tasks within their deadlines.

The tasks will be routed to the cloud if resources are not accessible in the fog layer.

#### 3.2.6. Task Scheduling Algorithm Design

Tasks are categorized into three types: normal tasks, moderate tasks, and critical tasks according to the patient’s condition in comparison with standard ECG intervals. All important tasks will be placed into a high-priority queue, while moderate tasks will be added to a separate queue, ensuring that all high-priority tasks are handled first. The suggested method executes a task categorization procedure depending on the patient’s status whenever a task comes from an edge device. Then, it puts the task into the appropriate queue according to its priority. After that, the dynamic task assignment algorithm assigns the task to the suitable layer. The Pseudo-Code of the proposed EEIoMT framework algorithm is shown in Algorithm 1.
**Algorithm 1.** Proposed EEIoMT Framework Algorithm **Input:** Tasks with patient’s condition attribute (Normal or Abnormal) History of heart attacks attribute (Yes, No) Number of tasks (T), Number of Fog nodes (F), Cloud **Output:** Optimize Energy Consumption and Latency **Begin:** 1. Incoming tasks T 2. **Foreach** task $t_{i}\in T$ 3. Call Task Classification Algorithm; 4.  Call Task Priority Algorithm; 5.  Call Dynamic Task Assignment Algorithm; 6. **End**

Therefore, a task classification algorithm was designed, as shown in Algorithm 2.
**Algorithm 2.** Task Classification Algorithm **Input**: Tasks with patient’s condition attribute (Normal or Abnormal)    History of heart attacks attribute (Yes, No) **Output:** Task Categorization (Normal, Moderate, or Critical) **Begin:** 1. Incoming tasks T 2. **Foreach** task $t_{i}\in T$ do 3.  **If** (patient condition is Normal) 4.   Categorize task t_i as Normal task 5.  **If** (patient condition is Abnormal) 6.   **Elseif** (heart attacks = yes) 7.    Categorize task t_i as Critical task 8.    **Else** 9.    Categorize task t_i as Moderate task 10.   **End** 11.  **End** 12. **End**

- Task priority algorithm

In the task priority algorithm, critical tasks are given high priority, moderate tasks are medium priority, and normal tasks are low priority. There are three queues for each task classification, and the tasks are arranged in each queue based on their deadlines. The EDF method is used to order the list of tasks by deadline. The assignment with the shortest deadline is completed first. When two jobs have the same deadline, the task is ordered by FCFS. Critical activities will be completed sooner as a result, helping to satisfy the deadline and reaction time requirements. The Pseudo-Code of the task priority algorithm is shown in Algorithm 3.
**Algorithm 3.** Task Priority Algorithm **Input:** Task Categorization (Normal, Moderate, or Critical) Number of tasks T **Output:** $critical\_tasks\;\left[t_{i},T];\;moderate\_tasks\;[t_{i},T];normal\_tasks\;[t_{i},T\right];$ **Begin:**
 1.  $critical\_tasks\;\left[t_{i},T\right]=null;$ 2.   $moderate\_tasks\;\left[t_{i},T\right]=null;$ 3.   $normal\_tasks\;\left[t_{i},T\right]=null;$ 4.   **Foreach**
$t_{i}\in T$
**do** 5.    **If** (task is normal) 6.     added to $normal\_tasks\;\left[t_{i},T\right]$; 7.    **Else if** (task is moderate) 8.     added to $moderate\_tasks\;\left[t_{i},T\right]\;$ 9.    **Else** 10.    added to $critical\_tasks\;\left[t_{i},T\right]$ 11.    **End** 12.  **End** 13.  **End**

- Dynamic task assignment algorithm

The Pseudo-Code of the dynamic task assignment algorithm is shown in Algorithm 4.
**Algorithm 4.** Dynamic Task Assignment Algorithm **Input:** $critical\_tasks\;\left[t_{i},T];\;moderate\_tasks\;[t_{i},T];normal\_tasks\;[t_{i},T\right];$ Number of fog node F, cloud **Output:** Task to be assigned in fog layer or cloud **Begin:** 1. **Foreach** $t_{i}\in critical\_tasks\;\left[t_{i},T\right]$) do 2.   Find the available fog nodes in the system; 3.    Calculate $Et\left(X_{ij}\right)$ based on Equation (3); 4.     Calculate $T_{r}t_{total}\left(X_{ij}\right)$ based on Equation (8); 5.     Calculate $RT\left(X_{ij}\right)$ based on Equation (10); 6.     Calculate $E_{total}\left(X_{ij}\right)$ based on Equation (16); 7.    $w_{E}=0;$ 8.    $w_{L}=1;$ 9.    Calculate the weight for all available fog node based on Equation (27); 10.    $Q_{F}\leftarrow$ Sort all available fog nodes by their weights with ascending order; 11.    **Foreach** $f_{j}\in Q_{F}$
**do** 12.     **If** $RT\left(X_{ij}\right)\leq dt_{i}$ 13.      **If** $E_{p}\left(X_{ij}\right)\leq \;E_{j}$ $\;\&\&\;TS_{i}\leq \;S_{j}$ 14.      Make the assignment based on Equations (21) and (26); 15.      $\;\;X_{ij}=1;$ 16.       break; 17.       **Else** 18.     Continue; 19.        **End** 20.     **End** 21.     **End** 22.     **If** $\;\;X_{ij}=0;$//No available fog nodes in fog layer 23.       Calculate $Et\left(X_{icloud}\right)$ based on Equation (4); 24.     Calculate $T_{r}t_{total}\left(X_{icloud}\right)$ based on Equation (9); 25.    Calculate $RT\left(X_{icloud}\right)$ based on Equation (11); 26.     Calculate $E_{total}\left(X_{icloud}\right)$ based on Equation (17); 27.      **If** $RT\left(X_{ij}\right)\leq dt_{i}$ 28.     Make the assignment $\;\;\;\;\;\;\;\;X_{icloud}=1;$ 29.      **End** 30.    **End** 31.      **End** 32.    **Foreach** $t_{i}\in moderate\_tasks\;\;\left[t_{i},T\right]$) **do** 33.      Find the available fog nodes in the system; 34.     Calculate $Et\left(X_{ij}\right)$ based on Equation (3); 35.     Calculate $T_{r}t_{total}\left(X_{ij}\right)$ based on Equation (8); 36.     Calculate $RT\left(X_{ij}\right)$ based on Equation (10); 37.     Calculate $E_{total}\left(X_{ij}\right)$ based on Equation (16); 38.      $w_{E}=0.5;$ 39.      $w_{L}=0.5;$ 40.     Calculate the weights for all available fog node based on Equation (27); 41.      $Q_{F}\leftarrow$ Sort all available fog nodes by their weight with ascending order; 42.    **Foreach** $f_{j}\in Q_{F}$
**do** 43.     **if** $RT\left(X_{ij}\right)\leq dt_{i}$ 44.       **if** $E_{p}\left(X_{ij}\right)\leq \;E_{j}$ $\&\&\;TS_{i}\leq \;S_{j}$ 45.      Make the initial assignment based on Equations (21) and (26); 46.      $\;\;X_{ij}=1;$ 47.       break; 48.      **Else** 49.     Continue; 50.      **End** 51.    **End** 52.     **End** 53.    **if** $\;\;X_{ij}=0;$//No available fog nodes in fog layer 54.      Calculate $Et\left(X_{icloud}\right)$ based on Equation (4); 55.      Calculate $T_{r}t_{total}\left(X_{icloud}\right)$ based on Equation (9); 56.      Calculate $RT\left(X_{icloud}\right)$ based on Equation (11); 57.      Calculate $E_{total}\left(X_{icloud}\right)$ based on Equation (17); 58.    **if** $RT\left(X_{ij}\right)\leq dt_{i}$ 59.     Make the assignment $\;\;\;\;\;\;\;\;X_{icloud}=1$; 60.   **End** 61.     **End** 62.    **End** 63.    **Foreach**
$t_{i}\in moderate\_tasks\;\;\left[t_{i},T\right]$) **do** 64.     Find the available fog nodes in the system; 65.    Calculate $Et\left(X_{ij}\right)$ based on Equation (3); 66.    Calculate $T_{r}t_{total}\left(X_{ij}\right)$ based on Equation (8); 67.    Calculate $RT\left(X_{ij}\right)$ based on Equation (10); 68.    Calculate $E_{total}\left(X_{ij}\right)$ based on Equation (16); 69.   $w_{E}=1;$ 70.   $w_{L}=0;$ 71.   Calculate the weight for all available fog node based on Equation (27); 72.   $\;Q_{F}\leftarrow$ Sort all available fog nodes by their weight with ascending order; 73.   **Foreach** $f_{j}\in Q_{F}$
**do** 74.    **if** $RT\left(X_{ij}\right)\leq dt_{i}$ 75.     **if** $E_{p}\left(X_{ij}\right)\leq \;E_{j}$ $\&\&\;TS_{i}\leq \;S_{j}$ 76.    Make the assignment based on Equations (21) and (26); 77.    $\;\;X_{ij}=1;$ 78.    break; 79.    **Else** 80.     Continue; 81.    **End** 82.     **End** 83.    **End** 84.   **if** $\;\;X_{ij}=0;$//No available fog nodes in fog layer 85.    Calculate $Et\left(X_{icloud}\right)$ based on Equation (4); 86.    Calculate $T_{r}t_{total}\left(X_{icloud}\right)$ based on Equation (9); 87.    Calculate $RT\left(X_{icloud}\right)$ based on Equation (11); 88.   Calculate $E_{total}\left(X_{icloud}\right)$ based on Equation (17); 89.    **if** $RT\left(X_{ij}\right)\leq dt_{i}$ 90.    Make the assignment $\;\;\;\;\;\;\;\;X_{icloud}=1$; 91.   **End** 92.    **End** 93.   **End**

#### 3.2.7. Scenario

Assume that a group of patients in a smart city is tracked in order to detect emergency circumstances such as arrhythmia. Each patient has an ECG sensor attached to his/her body that is connected to the ESP32 microcontroller. Assume that each group of ESP32 in a specific geographical area is linked to the one fog scheduler by a Wi-Fi connection. Each patient has a smartphone connected to the system, receiving important notifications from the system or the specialist doctor. ECG signals are collected in the ESP32 and analyzed periodically (every 8 s).

Case 1: If the outcome of the analysis shows that the patient’s condition is normal, the information will be aggregated, and the analysis will be performed every 24 s, for example, instead of 8 s, in order to limit the amount of energy used during the analysis. The patient’s health information and the results of the analysis will be delivered to the fog layer’s patient record database, which will be updated and stored.

Case 2: If the patient’s condition is abnormal, the result is sent to the fog scheduler through Wi-Fi. The proposed algorithm will first classify the tasks; if the patient has a previous history of heart attacks, their condition will be considered critical. It will be given high priority and executed at the appropriate fog node that fulfills the lowest possible latency. A sound alert will be sent to the smartphone to notify the patient’s family of their condition, and a notification will also be sent to the civil defense and the hospital.

Case 3: If the patient’s condition is abnormal and the patient has no history of heart attacks, their condition will be considered moderate. It will be given medium priority and executed at the available fog node. The patient’s data for the last 30 min will be sent to the hospital for further analysis. The specialist doctor will send a set of instructions for the patient to follow to avoid reaching a critical condition.

In all of the patient conditions, the patient’s health data will be delivered to the patient record database in the fog layer to update and store the data.

#### 3.2.8. Implementation

Using the Eclipse IDE environment (version 2021-03 (4.19.0), the suggested scheduling technique was implemented in the iFogSim2 simulation toolbox. The simulation was run on a real-world ECG dataset obtained from the University of California at Irvine’s Archive database (UCI) [42], which was utilized for cardiac arrhythmias. This database contains 452 heart rate samples, 279 attributes, and 16 classes. Some of the most important attributes are shown in Table 2. Table 3 shows the number and names of the classes, as well as the quantity of information in every class. Class 1 denotes a normal ECG, whereas classes 2 through 15 denote various classes of arrhythmia (abnormal). The rest of the unclassified ones are referred to as class 16.

In a 3000 × 2000 m area, six fog nodes were constructed and randomly placed. Every fog node had four connected IoT devices at first, with a coverage area of 500 m.

For the real-time implementation of our proposed framework, the configuration characteristics required for the cloud, proxy server, fog server, fog scheduler, and IoT devices are presented in Table 4.

The values of parameters used in the proposed EEIoMT framework are listed in Table 5.

## 4. Experimental Results and Discussion

In this part, the most important outcomes of the research are explained, discussed, and analyzed. Healthcare applications are time-sensitive and require real-time processing to monitor patients, and this is supported by the proposed framework. It also optimizes the QoS for home health monitoring systems by fog computing in terms of energy usage, latency, and network utilization. Since this system relates to human life, a delay in responding to critical tasks must have the lowest value, and this is what is provided in our proposed framework. Furthermore, caregivers can always control a patient’s state in real time and make the appropriate decision to save their life.

In this research, the simulation was run on a laptop with 8 GB of RAM, a 3.2 GHz Core i7 processor, 10th GEN DELL, a 500 GB HDD, 250 GB SSD, Windows 10 Pro, and a 64-bit operating system.

### 4.1. Experimental Results

The behavior evaluation of the suggested EEIoMT framework was performed against CHTM [31], LBS [29], and FNPA [30], which are high-level models, and outcomes are shown in this part. The following performance metrics are used in the performance evaluation:

1. Average energy consumption: This is the overall energy utilized by the entire system. It is measured by the amount of energy consumed while tasks are being transmitted and processed through any of the system elements, such as IoT devices, fog nodes, and the cloud. The energy consumption needs to be minimized. It is measured in joules.
2. Average latency: This is the time that it takes for the system to complete all of its tasks. In our system, the response time is adopted as the main criterion for lag, which is the total time required to transfer and perform tasks and return the results. As a result, when the reaction time is lowered, the latency is reduced as well. The latency needs to be minimized. It is measured in milliseconds (ms).
3. Network usage: In our research, the amount of data transferred and received by a certain user within a network in a given amount of time is referred to as bandwidth use. The more data exchanged, the greater the risk of clogging the network, and the more energy consumed by a single user. Typically, bandwidth is measured in bits per second and expressed as a bit rate (bps). In other words, it demonstrates how the proposed framework contributes to balancing network load while executing tasks. The network usage needs to be minimized. It is measured in kilobits per second (kbps).
4. CPU usage: The amount of work handled by a CPU or the number of processing resources used by a computer is referred to as “CPU use.” The volume and type of managed computing tasks determine how much CPU is used. Due to non-CPU resource needs, certain operations demand a lot of CPU time, while others require a lot less.

**Figure 4:**
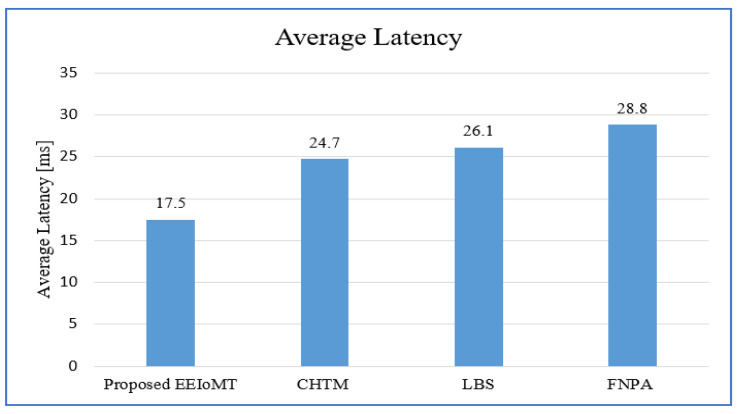
Comparison of Average Latency.

**Figure 5:**
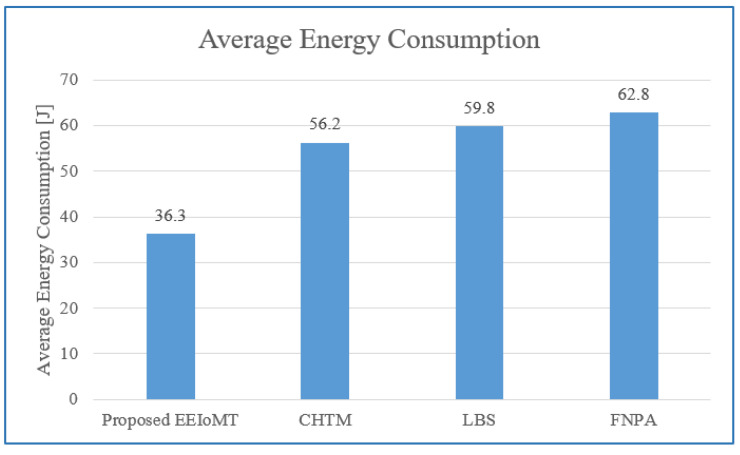
Comparison of Average Energy Consumption.

**Figure 6:**
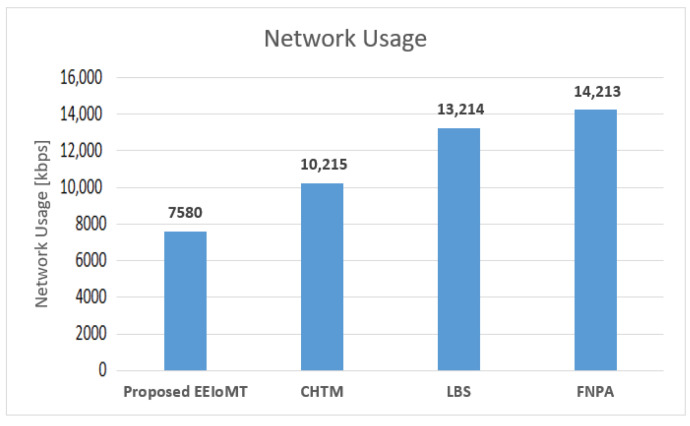
Comparison of Network Usage.

**Figure 7:**
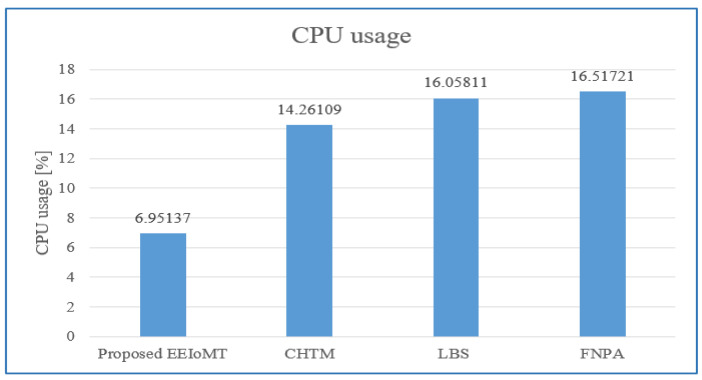
Comparison of CPU usage.

In the first experimental test, the number of fog nodes was set to 6, the number of VMs was 3, the number of patients was 24, and the number of tasks for each patient was 3. The results are the average of the 1000-time test. The outcomes for the behavior metrics of average latency, average energy consumption, network usage, and CPU usage are shown in Figure 4, Figure 5, Figure 6 and Figure 7, respectively.

The simulation results show that EEIoMT significantly reduces all of the performance metrics as compared to the CHTM, LBS, and FNPA models. This is due to the design of this framework, where the ECG sensor signals are analyzed near the sensor inside the ESP32 microcontroller; thus, the distance over which the signals are transmitted is greatly reduced, and therefore, the transmission time will be significantly reduced. If the result of the analysis indicates that the patient’s condition is normal, the data are aggregated and not sent directly to the fog layer, where in every given period of time, a report is sent with the patient’s data to be stored, and the previous data are updated in the fog layer and then sent to the cloud. However, if the patient’s condition is abnormal, the task with the abnormal condition will be sent to the fog layer, thus reducing the amount and size of data transmitted to the fog layer, which in turn reduces the latency, the amount of energy consumed during the transmission and processing of these tasks, and the amount of bandwidth consumed during the transmission of these tasks. Fog nodes are also used efficiently, and the load is distributed among them, which reduces the latency, energy consumption, and network usage that would result from giving one of the nodes a heavy burden and leaving the other idle. In the proposed framework, three algorithms are designed to improve the task execution process and reduce response time and energy consumption. The tasks are classified into normal, moderate, and critical tasks, and then each of them is given priority so that we ensure the execution of critical tasks in the least time and ensure that all other tasks are executed within their deadlines. The appropriate fog node is carefully selected to carry out the task by giving special weight to each node. In the case of critical tasks, the latency is given full importance, and thus, the fog node that achieves the least latency is chosen even if there is higher energy consumption. In the case of a moderate task, equal importance is given to energy consumption and latency, while when the task is normal, full importance is given to energy consumption while ensuring that tasks are executed within their deadline. In this way, the appropriate fog node is selected that achieves the best results depending on the type of task to be executed. In contrast, in the CHTM, LBS, and FNPA models, the sensor signals are sent to the fog layer, and thus, more time, energy, and bandwidth are consumed when transmitting and also when processing them in the fog nodes because the data size is much larger than the data that are transferred and processed through the proposed framework. Additionally, because the size of the tasks to be processed in our framework is small, they do not require high CPU usage.

It can be noticed that the proposed EEIoMT framework achieves the least latency, energy consumption, network usage, and CPU usage.

In the following experiments, the results were obtained by changing one parameter each time. These parameters are the number of patients, the number of fog nodes, the number of Virtual Machines (VMs), the number of tasks, and the percentage of the type of each task. All of the results are the average of the 1000-time test.

#### 4.1.1. Changing the Number of Patients

In this part of the experimental test, the number of fog nodes was set to 6, the number of VMs was 3, and the number of tasks for each patient was 3. As each patient has a fixed number of tasks, when the number of patients increases, the tasks will increase by a constant number of tasks for each patient, as shown in Table 6. The number of patients was changed to 36, 48, 60, and 72.

When the number of patients increases, the IoT devices connected to the fog layer will increase, and therefore, the tasks that are sent to the fog layer will increase. These tasks require more time and energy to transmit and process, and this increases latency and energy consumption. These tasks also consume more network bandwidth and CPU usage.

When the number of patients grows and there are not enough fog nodes with sufficient resources, the tasks are forwarded to the cloud. As a result of the increasing load on the cloud server, latency and network utilization increase.

**Figure 8:**
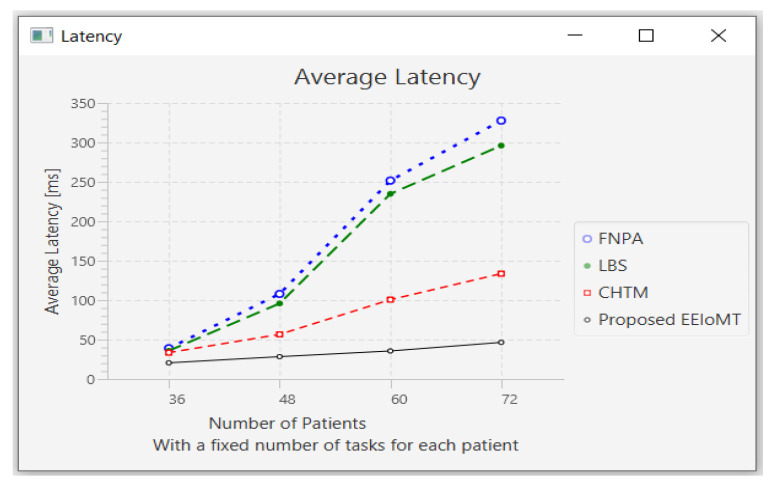
Average latency comparison when changing the number of patients.

**Figure 9:**
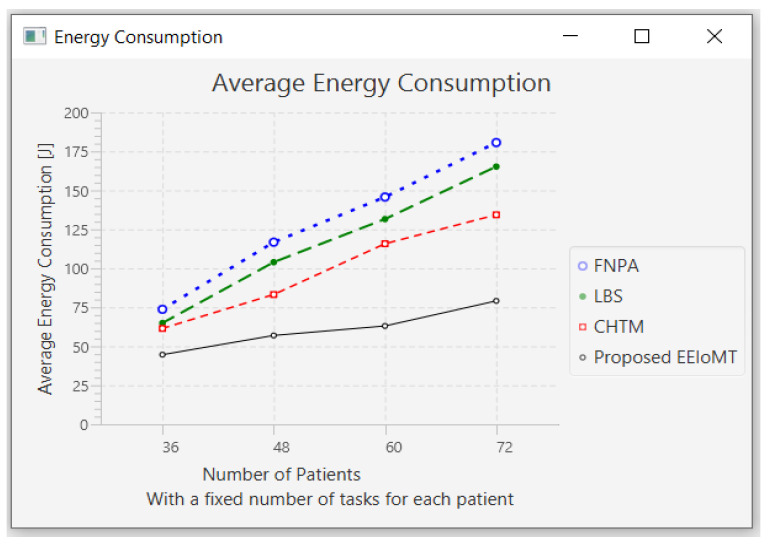
Average energy consumption comparison when changing the number of patients.

**Figure 10:**
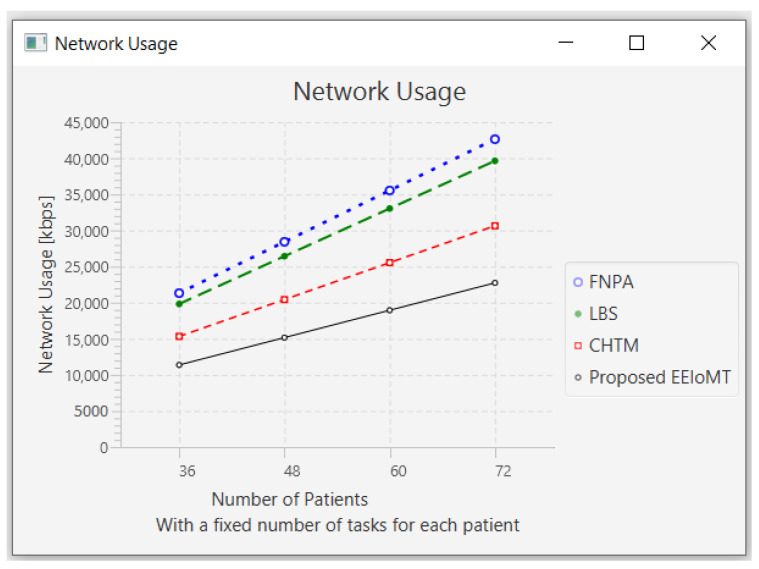
Network usage comparison when changing the number of patients.

**Figure 11:**
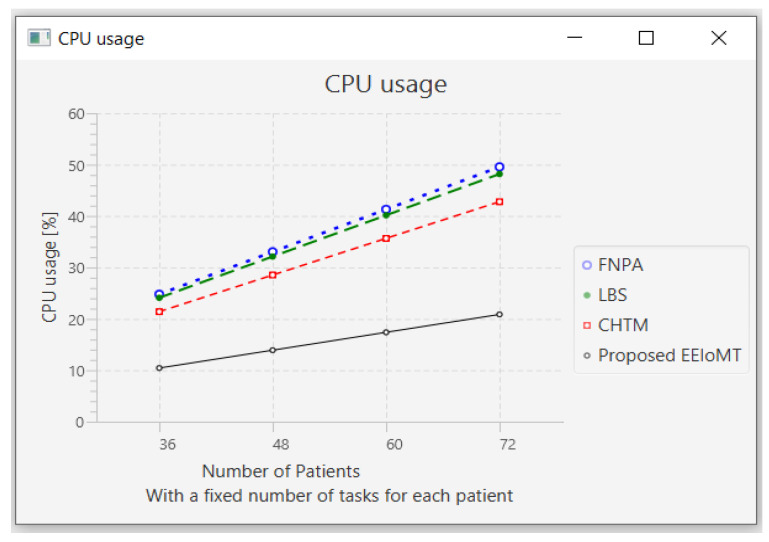
CPU usage comparison when changing the number of patients.

Figure 8, Figure 9, Figure 10 and Figure 11 illustrate the findings for the performance parameters of latency, energy consumption, network utilization, and CPU usage, respectively.

To allocate workload, FNPA chooses the fog node with the shortest distance and most resources (bandwidth, RAM, and CPU). When the number of users grows to the point where no more fog nodes with sufficient capacity are available, the tasks are routed to the cloud server. As a result of the increasing demand on the cloud server, latency increases. Likewise, each IoT device has only one fog node or BS in LBS, although it might be located in the coverage region of many BSs. In an effort to decrease latency, the IoT device chooses an appropriate fog node. The message is transmitted to IoT devices after each BS’s traffic and computing loads are iteratively calculated. The fog node’s communication and computation latency will be determined after the BS and fog nodes’ traffic and computing loads have been estimated, respectively. Each IoT device will verify the parameters before selecting the best BS in each iteration with the least amount of latency. When the number of IoT devices rises while the number of BSs remains the same, it is possible for many IoT devices to choose the same BS that achieves the least latency at the same time, and therefore, it receives an overload, and the latency will increase. Moreover, the selection of the suitable BS by IoT devices is inefficient, as there must be a scheduler for controlling these devices and selecting the most appropriate BS for them.

Prioritization is performed by Personal Agents (PAs) in the CHTM model before arrival tasks are designated to fog nodes. Then, in the fog nodes, utilizing Fog Node Agents (FNAs), all of the associated PAs are prioritized. The task scheduling module will decide whether to deal with incoming duties locally (if the task’s size is within the local node’s resources) or to send them to neighboring nodes (in case of the unattainability of local node resources). This module, in other words, provides three major options: executing locally, in a neighbor, or in the cloud.

#### 4.1.2. Changing the Number of Fog Nodes

In this part of the experimental test, the number of fog nodes was set to 24, the number of VMs was 3, and the number of tasks for each patient was 3. The number of fog nodes was changed to 8, 10, 12, and 14.

The simulation outcome indicates that as the number of fog nodes accumulates, latency, energy consumption, and network usage in all models decrease in varying proportions because when the number of fog nodes increases, there will be more options from which to choose the most suitable nodes to perform the tasks, and the possibility of sending tasks to the cloud will also be reduced, thus reducing latency and consuming less energy and less network bandwidth. There will also be more calculations for choosing the most suitable nodes to perform the tasks, and the execution of tasks will be distributed among these fog nodes, thus slightly decreasing CPU usage.

**Figure 12:**
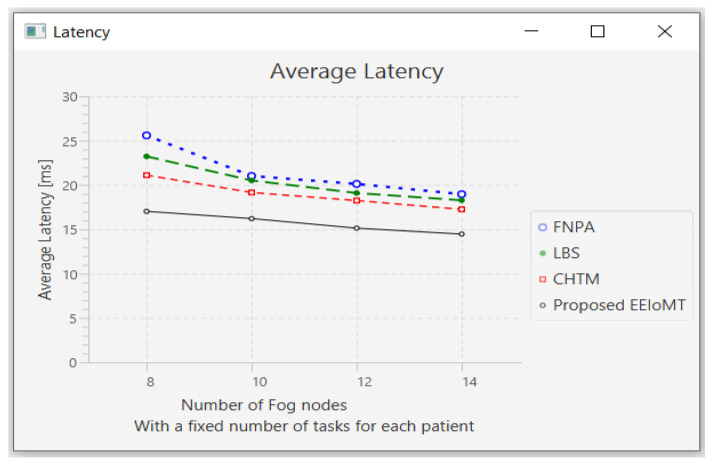
Average latency comparison when changing the number of fog nodes.

**Figure 13:**
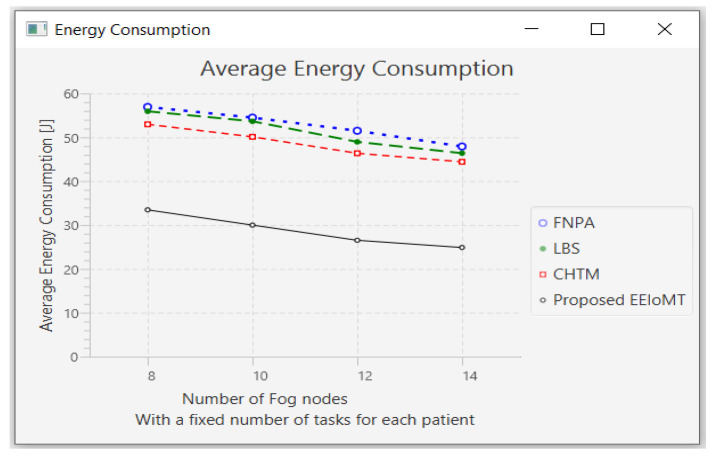
Average energy consumption comparison when changing the number of fog nodes.

**Figure 14:**
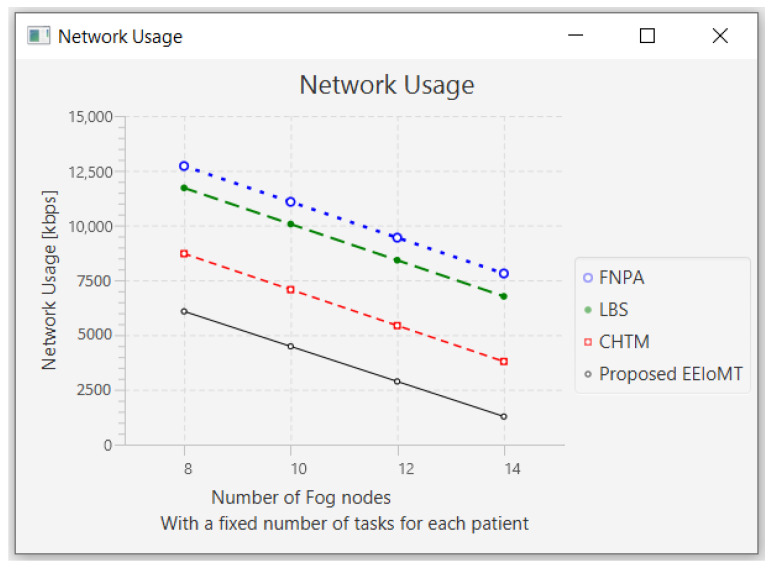
Network usage comparison when changing the number of fog nodes.

**Figure 15:**
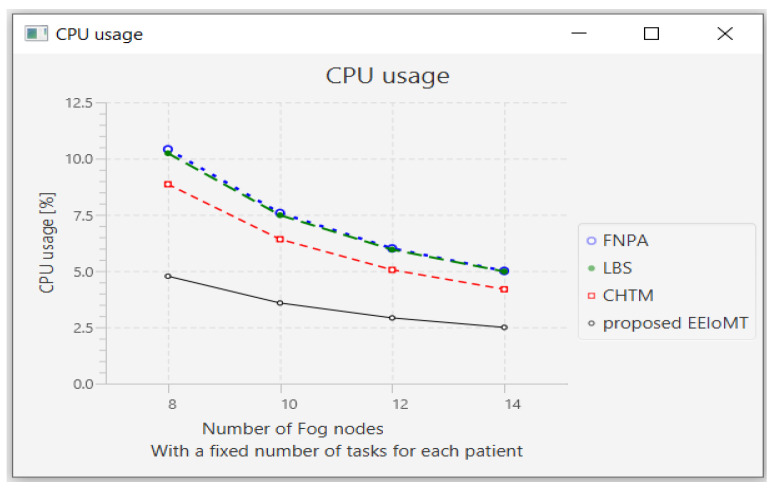
CPU usage comparison when changing the number of fog nodes.

The outcomes for the behavior metrics of latency, energy consumption, network usage, and CPU usage are shown in Figure 12, Figure 13, Figure 14 and Figure 15, respectively.

#### 4.1.3. Changing the Number of Virtual Machines

In this part of the experimental test, the number of fog nodes was set to 6, the number of patients was 24, and the number of tasks for each patient was 3. Then, the number of VMs was changed to 5, 7, 9, and 11.

**Figure 16:**
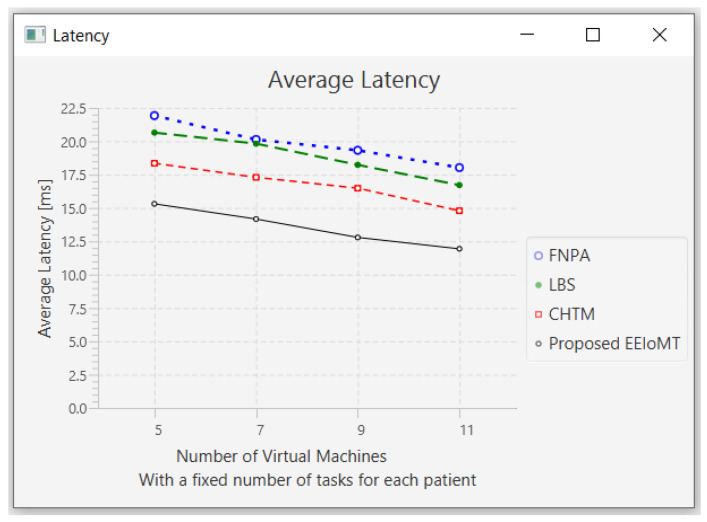
Average latency comparison when changing the number of VMs.

**Figure 17:**
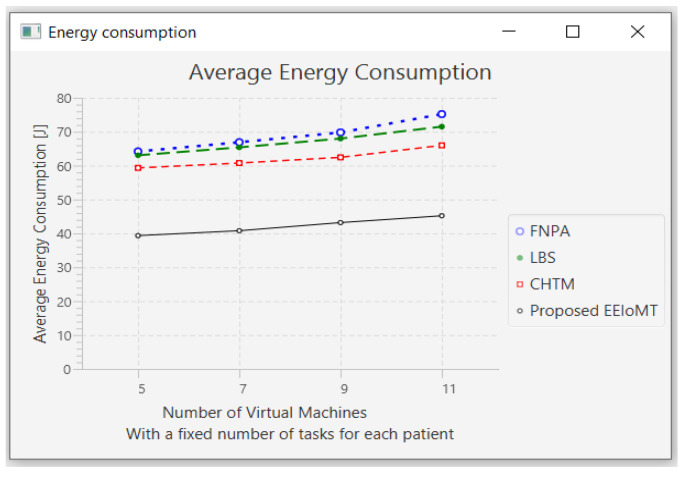
Average energy consumption comparison when changing the number of VMs.

**Figure 18:**
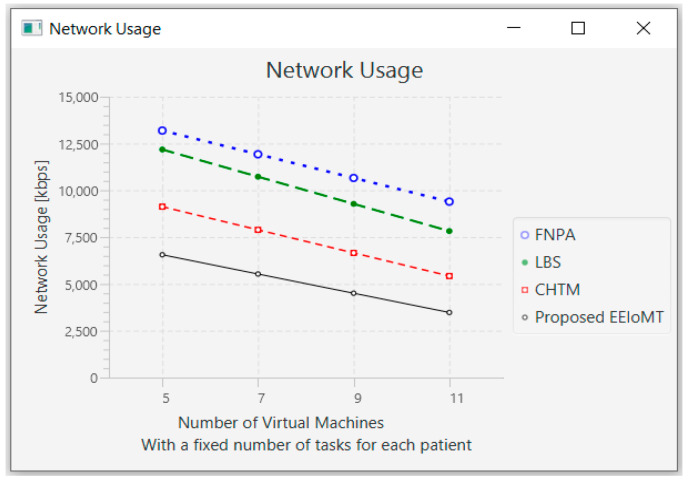
Network usage comparison when changing the number of VMs.

**Figure 19:**
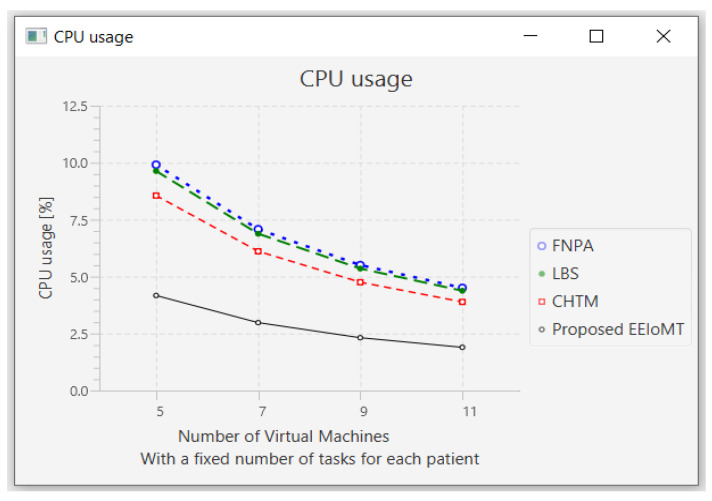
CPU usage comparison when changing the number of VMs.

When the number of VMs increases, most of the tasks will be executed in the fog layer, so the latency and the network usage will decrease, but the energy consumption will increase due to executing more tasks earlier, which requires more energy. The execution of tasks will also be distributed among these VMs, and thus, the CPU usage will decrease. Figure 16, Figure 17, Figure 18 and Figure 19 illustrate the findings for the performance measures of latency, energy consumption, network utilization, and CPU usage, respectively.

#### 4.1.4. Changing the Number of Tasks

In this part of the experimental test, the number of fog nodes was set to 6, the number of VMs was 3, and the number of patients was 24. Then, the number of tasks was changed to 120, 168, 216, and 264.

When the number of tasks rises, there is a rise in time and energy consumption to transmit and process these tasks. These tasks also need more processing in the CPU, so the CPU usage increases. Furthermore, these tasks consume more network bandwidth, so the network usage increases.

**Figure 20:**
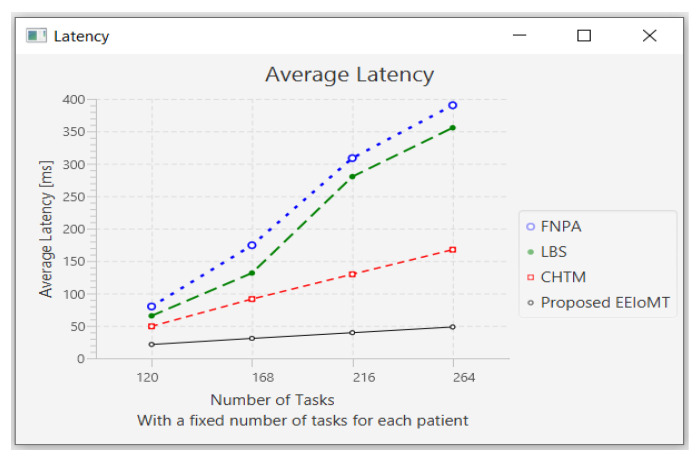
Average latency comparison when changing the number of tasks.

**Figure 21:**
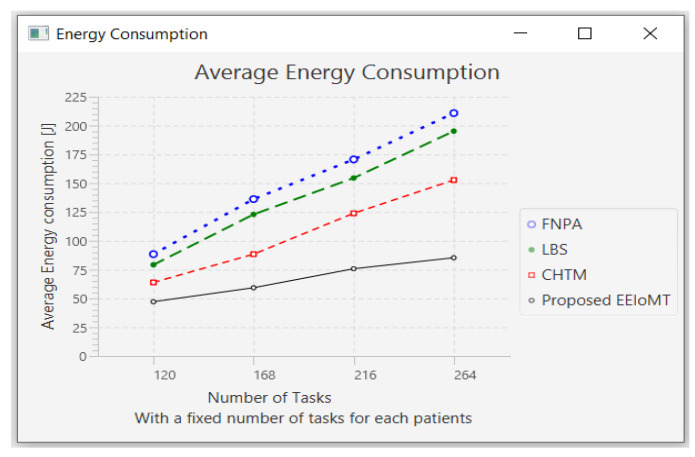
Average energy consumption comparison when changing the number of tasks.

**Figure 22:**
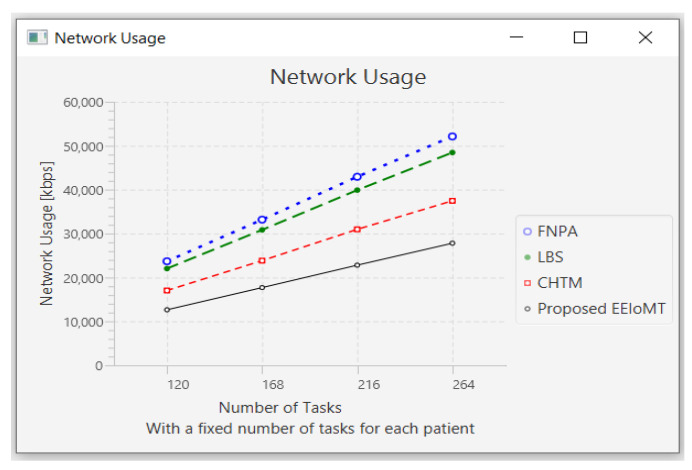
Network usage comparison when changing the number of tasks.

**Figure 23:**
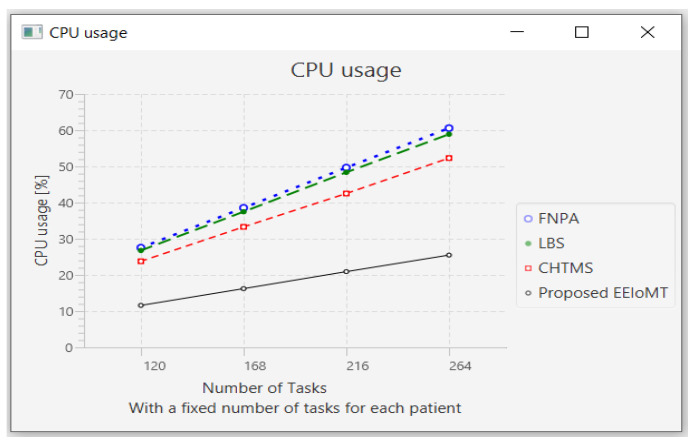
CPU usage comparison when changing the number of tasks.

The outcomes are presented in Figure 20, Figure 21, Figure 22 and Figure 23, which show performance metrics for latency, energy consumption, network usage, and CPU usage, respectively.

#### 4.1.5. Changing the Percentage of Each Type of Task

In this part of the experimental test, the number of fog nodes was set to 6, the number of VMs was 3, the number of patients was 24, and the number of tasks for each patient was 11. Then, the types of tasks were determined, namely, critical, moderate, or normal, and each type was given a certain percentage of the total number of tasks. The percentage of each type was changed to (70% normal, 20% moderate, 10% critical), (50% normal, 35% moderate, 15% critical), (30% normal, 40% moderate, 30% critical), and (10% normal, 50% moderate, and 40% critical).

The simulation results demonstrate that in our proposed EEIoMT, when the number of critical tasks increases, the energy consumption, network usage, and CPU usage are increased, but the latency is decreased because we are focused on minimizing latency. In this case, the fog nodes that achieve the least latency are selected, and when executing these tasks within the fog layer, the CPU usage and the bandwidth consumption will also increase.

It can be seen that in the proposed EEIoMT, when the percentage of normal tasks is 70%, the energy consumption is as low as possible because we are focused on minimizing energy while meeting their deadlines. In the other models, slight changes are observed in energy consumption, latency, network usage, and CPU usage because their tasks are not categorized and given priorities, as is implemented in our proposed framework, and therefore, changing the percentages of these tasks will not greatly change the values of any of the performance metrics in other models.

**Figure 24:**
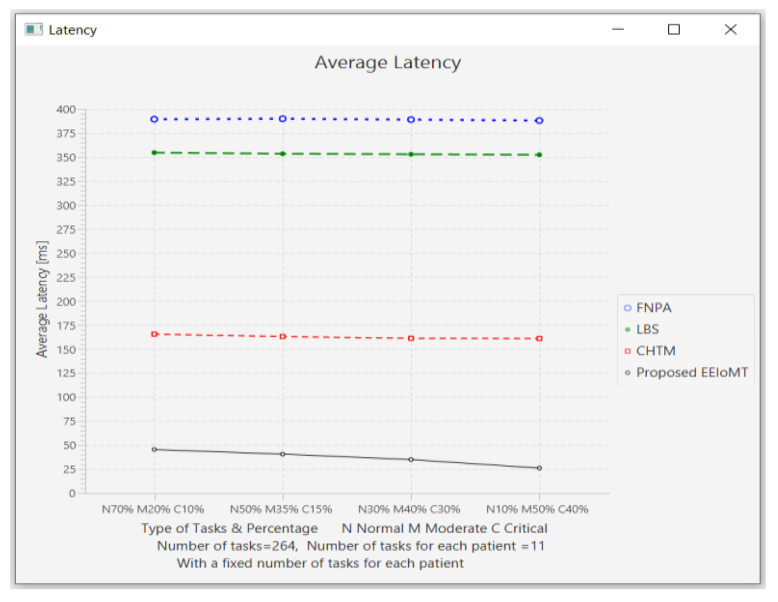
Average latency comparison when changing the percentage of each type of task.

**Figure 25:**
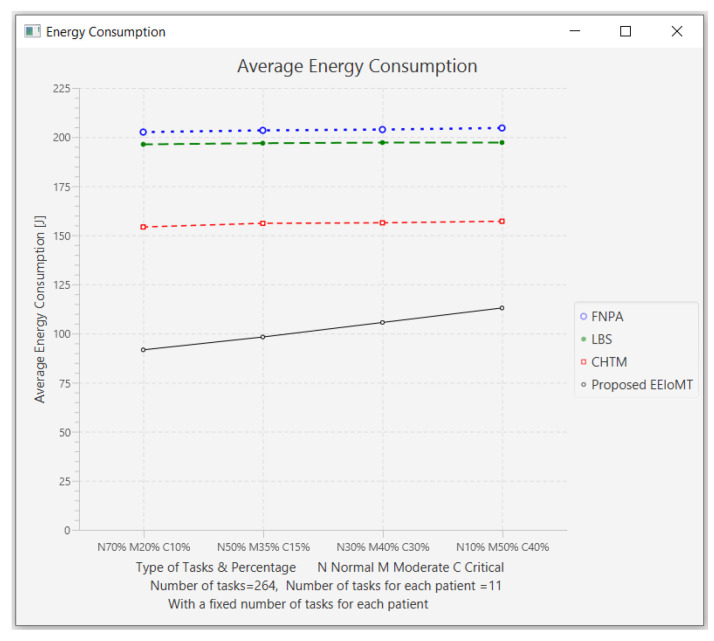
Average energy consumption comparison when changing the percentage of each type of task.

**Figure 26:**
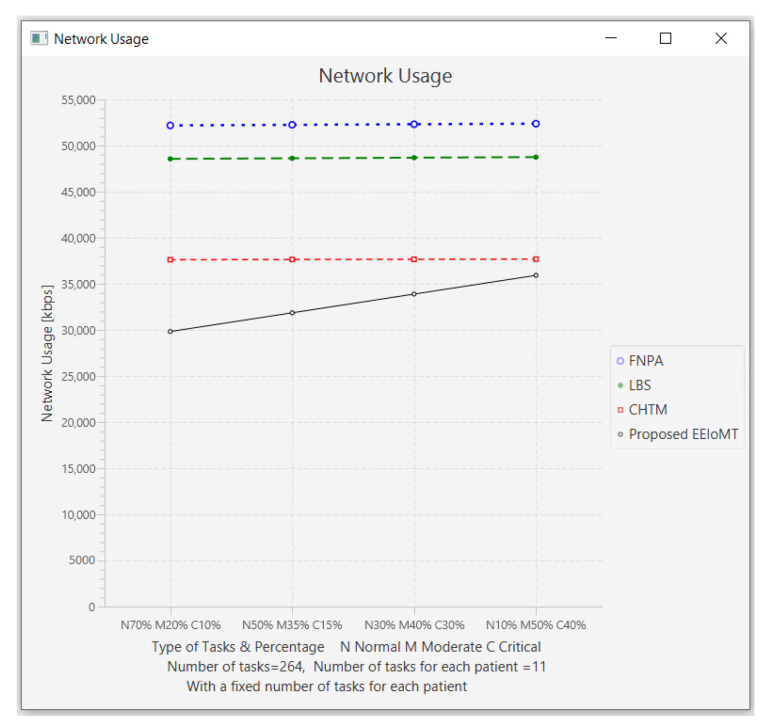
Network usage comparison when changing the percentage of each type of task.

**Figure 27:**
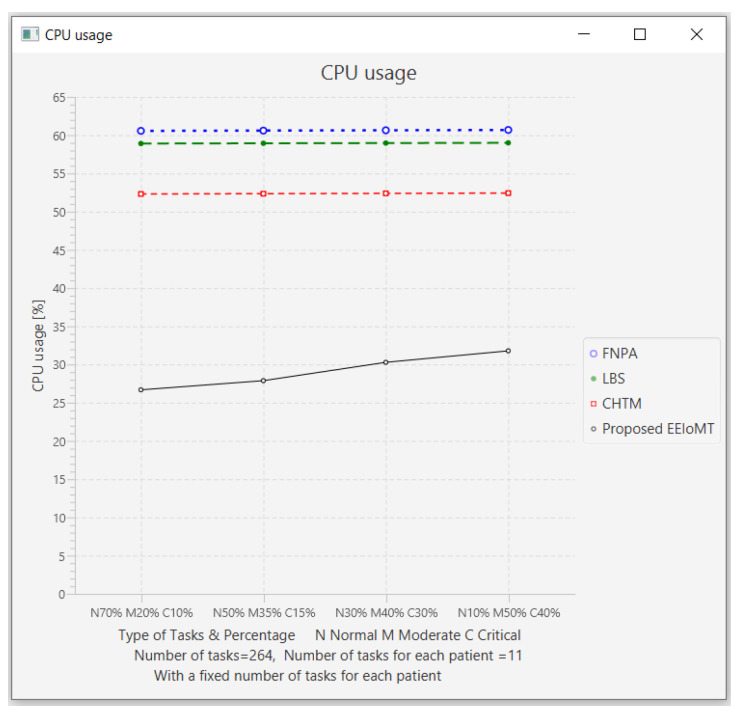
CPU usage comparison when changing the percentage of each type of task.

The results for the performance metrics of latency, energy consumption, network usage, and CPU usage are shown in Figure 24, Figure 25, Figure 26 and Figure 27, respectively.

In summary, the energy consumption in our work is composed of two portions: the amount of energy expended in completing a task to a computing node and the energy spent on executing the task. In time-sensitive applications such as health monitoring systems, latency must also be decreased. It can be observed that in the proposed EEIoMT, the latency increases and decreases depending on the type of task being performed. If most of them are critical tasks, the latency decreases, and if they are moderate or normal, the latency will be a little high. Furthermore, because only the cloud server is in charge of all data processing, using the cloud-only implementation of the architecture results in high network usage due to the expanded load of traffic on the cloud server. In contrast, in fog-based architectures such as EEIoMT, CHTM, LBS, and FNPA, every fog node is expected to handle and evaluate information streams acquired from its linked IoT devices, resulting in lower network use. Despite the scarce number of resources available at fog nodes, the burden is distributed among all fog nodes, resulting in decreased bandwidth usage. As a result, fog-based computing could be a great way to meet the QoS demands of real-time systems such as health monitoring. It is clear that the novel low-latency and energy-efficient task scheduling framework for the Internet of Medical Things in an edge–fog–cloud system can be used for many applications such as Vital Sign Monitoring [44], Smart-Contract-Aware Ethereum [45], hospitals and medical enterprises [46], Accelerating Edge Intelligence using Integrated Sensing [47], and a POMDP approach for age-of-information-based scheduling in multiuser uplinks with stochastic arrivals [48].

Regarding latency, energy consumption, and network usage, the suggested EEIoMT framework outperformed CHTM, LBS, and FNPA. The proposed fog-based health monitoring system will be able to process a high number of patient requests in a timely manner thanks to the proposed EEIoMT, which will assist patients, healthcare workers, and medical caregivers. People will be able to check their health condition at home and only need to see their doctor if their health status is critical, reducing the strain on clinics and hospitals.

## 5. Conclusions and Future Work

In recent years, the health field has received great attention due to the spread of the Coronavirus, which in turn has placed great pressure on hospitals and medical staff. Thus, the role and importance of healthcare applications in remote monitoring have greatly increased. In order to protect the elderly from this virus without direct interaction with medical staff who could transmit this virus to them, they can be monitored with the help of remote monitoring systems to prevent permanent stays in the hospital for monitoring. Therefore, the number of gadgets employed in these applications will rise, generating huge amounts of data. Using the cloud only causes delays while transferring information from sensors to the cloud and from the cloud to hospitals or caregivers. Fog computing, which sits between end-users (IoT devices) and cloud computing, was suggested by Cisco in 2012. Fog computing is not a replacement for cloud computing; rather, it mitigates cloud computing’s drawbacks and improves its efficiency. At the network’s edge, it also delivers storage and computational functions. Although fog computing is utilized in real-time healthcare applications, there are still limitations on response time, latency, and energy consumption. Although some researchers have proposed scheduling algorithms to overcome these limitations in a fog computing environment, there is still a gap in task scheduling regarding the time response and the amount of energy consumed. Most of the algorithms assign all incoming tasks coming from IoT devices to the nearest fog node without giving them priorities and without regard for the load on the fog nodes.

This research mitigated these limitations by proposing an EEIoMT framework. This framework consists of three algorithms for efficient task scheduling to ensure that critical tasks are executed in the shortest possible time within the deadline while balancing energy consumption when processing other tasks. In our architecture, ECG sensors are used to monitor heart health at home in a smart city. The ECG sensors continuously send the sensed data to the ESP32 through BLE. The ECG signals are analyzed in the ESP32 microcontroller, which is also connected to the fog scheduler via Wi-Fi connection to send the analysis results data (tasks). The appropriate fog node is carefully selected to perform the task by giving each node a special weight based on the expected amount of energy consumed and latency in performing the task and choosing the node with the lowest weight.

The proposed algorithm was assessed utilizing the iFogSim2 simulator. The simulation outcomes show that our framework, in comparison with the CHTM model, decreases the latency by 59%, energy consumption by 61%, and network usage by 57%. When compared with LBS, it reduces the latency by 60%, the consumption of energy by 62%, and the network usage by 64%. In comparison with FNPA, it reduces the latency by 62%, energy consumption by 63%, and network usage by 65%. The results indicate that the suggested framework has superior performance in decreasing energy consumption, latency, and network usage when contrasted to CHTM, LBS, and FNPA.

In the future, an enhancement to the framework is needed in order to process more types of vital signs simultaneously. In addition, the proposed framework will be applied in other real-time applications in addition to healthcare applications. Furthermore, the suggested algorithm needs to be tested on larger and more diverse data with real equipment if possible.

## Figures and Tables

**Table 1:** Normal values of waves and intervals of ECG signal [39].

Feature	Amplitude (Millivolts)	Duration (Milliseconds)
P wave	0.1–0.2	60–80
PR interval		120–200
PR segment		50–120
RR interval		600–1200
QRS complex	1	80–120
ST segment		100–120
ST interval		320
QT interval		400–430
T wave	0.1–0.3	120–160
TP segment		380–400
Heart rate per minute		60–100 bmp

**Table 2:** The UCI database attribute information [42].

Attribute	Description
Age	Age in years
Sex	Sex (0 = male, 1 = female)
Height	Height in centimeters (cm)
Weight	Weight in kilograms (kg)
QRS duration	Average QRS duration in milliseconds.
P-R interval	Average time interval between the start of waves P and Q in milliseconds
Q-T interval	Average time interval between the start of wave Q and end of wave T in milliseconds
T interval	Average time interval of wave T in milliseconds
P interval	Average P wave distance in milliseconds
Heart rate	Number of heart beats per minute

**Table 3:** The UCI database arrhythmia classes [43].

Number of Class	Name of Class	Amount of Data in Class
1	Normal	245
2	Ischemic changes (coronary artery diseases)	44
3	Old anterior myocardial infarction	15
4	Old inferior myocardial infarction	15
5	Sinus tachycardia	13
6	Sinus bradycardia	25
7	Ventricular premature contraction	3
8	Supraventricular premature contraction	2
9	Left bundle branch block	9
10	Right bundle branch block	50
11	Degree atrioventricular block	0
12	Degree AV block	0
13	Degree AV block	0
14	Left ventricular hypertrophy	4
15	Atrial fibrillation or flutter	5
16	Others	22

**Table 4:** Value of parameters used for cloud- and fog-based implementations [24].

Parameter Name and Unit	Cloud	Proxy	Fog Server	Fog Scheduler	IoT Device
CPU length (MIPS)	44,800	2800	2800	2800	500
Random access memory (MB)	40,000	4000	4000	4000	2048
Uplink bandwidth (MB)	100	10,000	10,000	10,000	10,000
Downlink bandwidth (MB)	10,000	10,000	10,000	10,000	270
Level	0	1	2	2	3
Rate per MIPS	0.01	0.0	0.0	0.0	0.0
Busy power (Watt)	16 × 103	107.339	107.339	107.339	87.53
Idle power (Watt)	16 × 83.25	83.4333	83.4333	83.4333	82.44

**Table 5:** Value of parameters used in proposed EEIoMT framework.

Parameter	Value
Network size	3000 × 2000 m
Number of fog nodes	6
Number of VMs	3
Number of tasks for each patient	3
Number of patients	24
Network length (bytes)	22,000
λ	1224.78 mJ/s
μ	3.7 (watt)

**Table 6:** Number of tasks when increasing the number of patients.

Number of Patients	Number of Tasks	Number of Tasks for Each Patient
36	108	3
48	144	3
60	180	3
72	216	3

## Data Availability

In this research, the data were taken from the Cleveland dataset, which is freely available online at http://archive.ics.uci.edu/mL/datasets/arrhythmia (accessed on 16 January 2022).

## Nomenclature

T	Set of tasks
n	Number of tasks
$t_{i}$	Task i∈T
$TS_{i}$	Task size of task $t_{i}$
$TL_{i}$	Task length of task $t_{i}$
$\;type_{i}$	The type of task $t_{i}$, which is normal, moderate, or critical
$dt_{i}$	The deadline of task $t_{i}$ to be respected for completing the task
F	Set of fog nodes
m	Number of fog nodes in the system
$f_{j}$	Fog node j∈F
$S_{j}$	Storage capacity of fog node $f_{j}$
$CC_{j}$	Computing capacity of fog node $f_{j}$
$E_{j}$	Total battery capacity of $Fog\;node\;\;f_{j}$
$X_{ij}$	A binary variable determining whether $t_{i}$ is assigned to $f_{j}$ or not
$X_{icloud}$	A binary variable determining whether $t_{i}$ is assigned to the cloud or not
$Et\left(X_{ij}\right)$	Execution time of processing task $t_{i}$ on fog node $f_{j}$
$Et\left(X_{icloud}\right)$	Execution time of processing task $t_{i}$ in the cloud
$CC_{cloud}$	Computing capacity of the cloud
$T_{r}t\left(FS\right)$	The transmission time of task $t_{i}$ to the fog schedule
BW	The transmission rate of the task to be sent from one node to the other
$T_{r}t\left(X_{ij}\right)$	The transmission time of task $t_{i}$ from the fog scheduler to fog node $f_{j}$
$T_{r}t\left(X_{icloud}\right)$	The transmission time of task $t_{i}$ from the fog scheduler to the cloud
$T_{r}t_{total}\left(X_{ij}\right)$	The total transmission time of tasks from the ESP32 or smartphone to fog node $f_{j}$
$T_{r}t_{total}\left(X_{icloud}\right)$	The total transmission time of tasks from the ESP32 or smartphone to the cloud
$RT\left(X_{ij}\right)$	The response time of tasks $t_{i}$ that are processed in fog node $f_{j}$
$RT\left(X_{icloud}\right)$	The response time of tasks $t_{i}$ that are processed in the cloud
$E_{tr}\left(X_{ij}\right)$	The energy required to transmit task $t_{i}$ to fog node $f_{j}$
$E_{tr}\left(X_{icloud}\right)$	The energy required to transmit task $t_{i}$ to the cloud
λ	Constant related to the wireless interface
$E_{p}\left(X_{ij}\right)$	The energy consumption for processing task $t_{i}$ on fog node $f_{j}$
$E_{p}\left(X_{icloud}\right)$	The energy consumption for processing task $t_{i}$ in the cloud
μ	The coefficient denoting the energy consumption per CPU cycle
$E_{total}\left(X_{ij}\right)$	The total energy is calculated by a combination of processing energy and transmission energy for fog
$E_{total}\left(X_{icloud}\right)$	The total energy is calculated by a combination of processing energy and transmission energy for the cloud
$X_{assign}$	The weight assigned to fog node $f_{j}$
$N_{ij}^{WSM-score}$	The QoS score of $X_{ij}$
$w_{E}$	The weight factors related to the energy consumption in a selected node
$w_{L}$	The weight factors related to the latency in a selected node
CT	The completion time
RU	Resource Utilization
